# Construction of a (NNN)Ru-Incorporated Porous Organic Polymer with High Catalytic Activity for β-Alkylation of Secondary Alcohols with Primary Alcohols

**DOI:** 10.3390/polym14020231

**Published:** 2022-01-07

**Authors:** Yao Cui, Jixian Wang, Lei Yu, Ying Xu, David J. Young, Haiyan Li, Hongxi Li

**Affiliations:** 1 College of Chemistry, Chemical Engineering and Materials Science, Soochow University, Suzhou 215123, China; cuiyao7851@163.com (Y.C.); 18794315203@163.com (J.W.); 2 Analysis and Testing Center, Soochow University, Suzhou 215123, China; yulei@suda.edu.cn (L.Y.); xuying@suda.edu.cn (Y.X.); lihaiyanyan@suda.edu.cn (H.L.); 3 College of Engineering, Information Technology and Environment, Charles Darwin University, Brinkin 0909, Australia; david.young@cdu.edu.au

**Keywords:** heterogeneous catalysis, porous organic polymer, Ru NNN pincer complex, β-alkylation of secondary alcohols

## Abstract

Solid supports functionalized with molecular metal catalysts combine many of the advantages of heterogeneous and homogeneous catalysis. A (NNN)Ru-incorporated porous organic polymer (POP-bp/bbpRuCl_3_) exhibited high catalytic efficiency and broad functional group tolerance in the C–C cross-coupling of secondary and primary alcohols to give β-alkylated secondary alcohols. This catalyst demonstrated excellent durability during successive recycling without leaching of Ru which is ascribed to the strong binding of the pincer ligands to the metal ions.

## 1. Introduction

The construction of carbon–carbon bonds is fundamental to organic synthesis [1,2]. Alkyl halides were the workhorse of this transformation last century, but replacing these relatively toxic and wasteful electrophiles has been an imperative in recent years [3,4,5]. Transition-metal-catalyzed α- or β-alkylation of carboxyl complexes, nitriles and secondary alcohols using alcohols as alkylating agents generates only hydrogen and/or water as waste [6,7,8]. Metal-catalyzed heterocoupling reactions of secondary and primary alcohols proceeds through controllable dehydrogenation–condensation–hydrogenation steps, selectively affording α,β-unsaturated ketones, α-alkylated ketones or β-alkylated secondary alcohols [9,10,11,12,13]. Various molecular complexes based on Ru [14,15,16,17], Ir [18,19,20,21], Co [22,23,24,25], Ni [26,27,28,29,30], and Mn [31,32,33,34,35,36] have been developed for these transformations. However, these are homogeneous catalysts and separation and recycling is an impediment to their large-scale industrial use. Some heterogeneous catalysts, including IrO_2_/Fe_3_O_4_ [37], Ni/Ca_x_Mg_y_O [38], SBA-15-supported Ir/NHC complex [39], Pt–Sn/γ-Al_2_O_3_ [40], DMF-stabilized Ir nanoclusters [41] have been developed for the cross-alkylation of alcohols to give β-alkylated secondary alcohols. However, these systems suffer from the need for relatively high temperatures, and/or long catalytic reaction time, intricate formulations.

Porous organic polymers (POPs) with high stability, low skeleton density, tunable porous structures and easy synthesis are an intriguing platform for incorporating homogeneous metal/ligand molecular catalysts into heterogeneous supports [42,43,44,45]. One particular advantage of POPs is the range of possible chemical functionality within the porous framework. Phosphines, pyridines, bipyridyl/phenanthroline derivatives, porphyrins, carbenes, and salen have been introduced into the skeleton of POPs [46,47,48,49]. Functionalized POPs with metal ions or nanoparticles have demonstrated excellent catalytic performance for the Suzuki-Miyaura coupling reaction [50], Heck reaction [51], Ullmann coupling reaction [52], CO_2_ conversion into cyclic carbonates [53,54], alkyne carboxylation [55,56,57], photocatalytic hydrogen evolution [58], the reduction of nitroarenes [59,60,61] and the hydroformylation of higher olefins [62]. In the present work, we have incorporated 2,6-bis(benzimidazo-1-yl)pyridine (bbp) into a POP through a simple one-pot Scholl co-coupling polymerization with biphenyl to give NNN pincer based POP (POP-bp/bbp). RuCl_3_-incorporated POP-bp/bbp (POP-bp/bbpRuCl_3_) displayed high catalytic performance, selectivity and recyclability for the β-alkylation of secondary alcohols with primary alcohols.

## 2. Materials and Methods

### 2.1. Materials

2,6-Bis(benzimidazo-1-yl)pyridine (bbp) was prepared according to a published procedure [63]. All chemicals were commercially available and used as received without further purification. Biphenyl, 2,6-pyridinedicarboxylic acid, o-phenylenediamine, phenylmethanol, (4-chlorophenyl)methanol, (4-(trifluoromethyl)phenyl)methanol, p-tolylmethanol, (4-methoxyphenyl)methanol, (4-(tert-butyl)phenyl)methanol, o-tolylmethanol, (2-methoxyphenyl)methanol, (3-methoxyphenyl)methanol, m-tolylmethanol, (3-chlorophenyl)methanol, naphthalen-1-ylmethanol, benzo[d][1,3]dioxol-5-ylmethanol, thiophen-2-ylmethanol, 1-phenylethan-1-ol, 1-(4-chlorophenyl)ethan-1-ol, 1-(4-bromophenyl)ethan-1-ol, 1-(p-tolyl)ethan-1-ol, 1-(4-methoxyphenyl)ethan-1-ol, 1-(2-chlorophenyl)ethan-1-ol, 1-(2-methoxyphenyl)ethan-1-ol, 1-(o-tolyl)ethan-1-ol, 1-(3-methoxyphenyl)ethan-1-ol, 1-(m-tolyl)ethan-1-ol, 1-(naphthalen-2-yl)ethan-1-ol, RuCl_3_·xH_2_O, CDCl_3_ were purchased from J&K. Toluene, chloroform, methanol, tetrahydrofuran, N,N-dimethylformamide, dimethyl sulfoxide, petroleum ether, and ethyl acetate were from Shanghai Titan Technology Co., Ltd. (Shanghai, China). Orthophosphoric acid, hydrochloric acid, aluminum chloride, potassium hydroxide, potassium tert-butoxide, cesium carbonate, sodium hydroxide, cesium hydroxide got from Sinopharm Chemical Reagent Co. Ltd. (Shanghai, China).

### 2.2. Synthesis of Porous Organic Polymer (POP)-bp/bbp

Under a N_2_ atmosphere, 2,6-bis(benzimidazo-1-yl)pyridine (bbp) (0.311 g, 1 mmol) and biphenyl (0.154 g, 1 mmol) were dissolved in anhydrous chloroform (20 mL), followed by the addition of anhydrous aluminum chloride (10 mmol, 1.33 g) with vigorous stirring at 58 °C. After 24 h, the reaction mixture was cooled to ambient temperature, filtered and the solid product was washed with methanol, HCl-H_2_O (*v*/*v* = 1:1), H_2_O, CH_2_Cl_2_ and EtOH, followed by washing with methanol in a Soxhlet thimble for 72 h. The polymer was then dried in a vacuum oven at 100 °C for 24 h to give a brown solid. Yield: 0.418 g (90%). Elemental analysis: calculated: C, 82.66; H, 4.95; N, 15.05. Found: C, 73.86; H, 4.19; N, 13.42.

### 2.3. Synthesis of POP-bp/bbpRuCl_3_

Under a N_2_ atmosphere, a mixture of POP-bp/bbp (0.2 g), RuCl_3_·xH_2_O (0.017 g), and anhydrous ethanol (25 mL) was introduced into a round-bottom flask (100 mL). The resulting mixture was refluxed for 12 h. After this period, the brown solid POP-bp/bbpRuCl_3_ was separated by centrifugation, washed sequentially with ethanol and ethyl ether, and then dried under vacuum.

### 2.4. Typical Procedure for Syntheses of β-Alkylated Secondary Alcohols

Under an N_2_ atmosphere, a mixture of secondary alcohol (1 mmol), primary alcohol (1.2 mmol), POP-bp/bbpRuCl_3_ (20 mg, 0.6 mol% Ru), KOH (0.5 mmol) and toluene (2 mL) was added into a 15 mL sealed tube equipped with a stirring bar. The reaction mixture was heated to 130 °C for 12 h. After cooling to ambient temperature, the catalyst was separated by centrifugation and washed with ethanol and diethyl ether. The catalyst was then dried in a vacuum at 60 °C for 2 h to give recycled catalyst for the next run. The organic layers were combined and dried over anhydrous Na_2_SO_4_ and concentrated under reduced pressure. The crude product was purified by flash column chromatography using petroleum ether and ethyl acetate as the eluent.

### 2.5. Characterizations

The analytical instruments employed in this work are as described in our previous article [57,64,65], unless otherwise noted. ^1^H and ^13^C NMR spectra were recorded at ambient temperature on a Varian UNITY plus-400 spectrometer. Solid-state cross-polarization magic angle spinning ^13^C NMR measurements were carried out on Bruker Avance III/WB solid-state NMR spectrometer operating at 400 MHz equipped with a standard 4 mm magic angle spinning double resonance probe head. Powder X-ray diffraction patterns were collected on a PANalytical Aeris diffractometer (Cu-Ka). Infrared spectra were recorded on a Varian Scamiter-1000 spectrometer. The thermal stability of materials was evaluated using thermogravimetric analysis (Perkin-Elmer Pyrisl) under a nitrogen atmosphere. Scanning electron microscopy (SEM) images and energy dispersive X-ray spectroscopy spectra were obtained using a HITACHI S-4700 cold field-emission SEM. Transmission electron microscopy (TEM) was performed on a FEI Tecnai G20 electron microscope operating at 200 kV. Annular dark-field scanning TEM (ADF-STEM) was performed on a FEI Tecnai F20 electron microscope operating at 200 kV, equipped with Genesis EDS detector. X-ray photoelectron spectra were recorded on an X-ray photoelectron spectrometer (AXIS Ultra DLD) and binding energies were referenced to C 1s at 284.7 eV from hydrocarbon to compensate for possible charging effects.

## 3. Results and Discussion

### 3.1. Subsection Synthesis and Characterization of POP-bp/bbpRuCl_3_

**Scheme 1:**
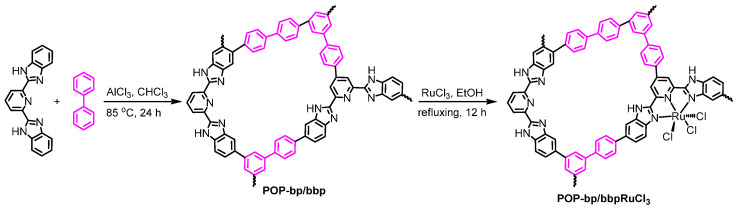
Synthesis of porous organic polymer (POP)-bp/bbp and POP-bp/bbpRuCl_3_.

**Figure 1:**
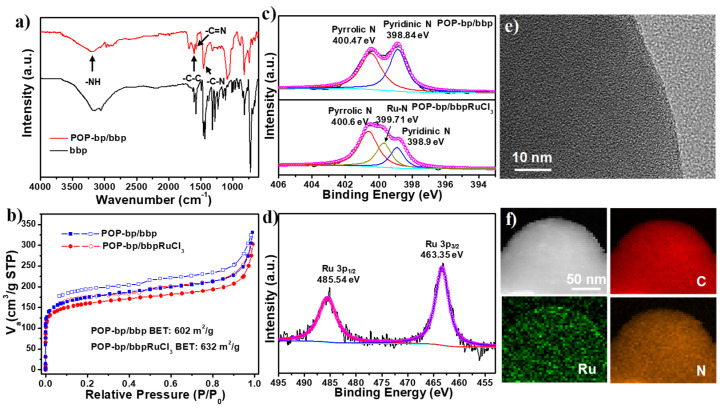
(**a**) Fourier transform infrared (FT-IR) spectra of POP-bp/bbp and POP-bp/bbpRuCl_3_. (**b**) N_2_ sorption isotherm for POP-bp/bbp and POP-bp/bbpRuCl_3_ at 77 K. (**c**) N1s X-ray photoelectron spectroscopy (XPS) spectra of POP-bp/bbp and POP-bp/bbpRuCl_3_. (**d**) Ru 3p XPS spectra of POP-bp/bbpRuCl_3_. (**e**) High-resolution transmission electron microscopy (HR-TEM) image of POP-bp/bbpRuCl_3_. (**f**) Energy-dispersive X-ray spectroscopy elemental mapping of POP-bp/bbpRuCl_3_.

POP-bp/bbp was synthesized by the AlCl_3_-promoted Scholl reaction of bbp and biphenyl (bp) in CHCl_3_ at 58 °C (Scheme 1). The resulting brown polymer was insoluble in common organic solvents such as tetrahydrofuran (THF), methanol (MeOH), N,N-dimethylformamide (DMF) and dimethyl sulfoxide (DMSO). Thermogravimetric analysis (TGA) showed that POP-bp/bbp was stable at temperatures up to 300 °C (Figure S1). The ^13^C NMR solid-state spectrum of POP-bp/bbp contained signals at δ = 148, 140 and 138 ppm ascribed to the carbons on pyridine and imidazole rings, and a broad signal at δ = 125 ppm assigned to the other aromatic carbons (Figure S2). Scanning electron microscopy (SEM) (Figure S3) and transmission electron microscopy (TEM) (Figure S4) images showed that the as-prepared polymer POP-bp/bbp was amorphous with particles of irregular shape and size. The energy-dispersive X-ray (EDX) elemental mapping images (Figure S5) indicated the homogenous distribution of C and N, indicating homogeneous distribution of bbp monomer. The FT-IR spectrum (Figure 1a) contained absorptions at 1600, 1573, and 1460 cm^−1^ attributed to the C−C, C=N and C−N stretching vibrations, respectively. In addition, the band at 3185 cm^−1^ were assigned to the −NH, originating from the imidazole moiety.

POP-bp/bbp bearing NNN pincer groups was treated with RuCl_3_·xH_2_O in refluxing EtOH under N_2_ for 12 h to give Ru-metalated POP-bp/bbp (POP-bp/bbpRuCl_3_). The porosity of POP-bp/bbp and POP-bp/bbpRuCl_3_ was assessed by N_2_ adsorption and desorption analyses at 77.3 K. Absorption isotherms (Figure 1b) exhibited a type I adsorption isotherm with steep N_2_ uptake at low relative pressure (P/P_0_ < 0.1), indicating abundant micropores in the polymer structure. A slight hysteresis loop with a small rise in N_2_ uptake at higher pressures was attributed to the presence of mesopores. The Brunauer−Emmett−Teller (BET) surface areas of POP-bp/bbp and POP-bp/bbpRuCl_3_ were calculated to be 632 and 602 m^2^ g^−1^, respectively. The pore-size distribution of POP-bp/bbp and POP-bp/bbpRuCl_3_ were estimated with the aid of non-local density functional theory modeling to be centered around 6−10 Å (Figure S7). Inductively coupled plasma optical emission spectrometry (ICP-OES) of POP-bp/bbpRuCl_3_ indicated 3.11 wt % Ru loading. The powder X-ray diffraction (PXRD) patterns confirmed that both the polymer and POP-bp/bbpRuCl_3_ were amorphous (Figure S8).

Ruthenium ion interaction with the NNN pincer moieties were observed by XPS (Figures S9 and S10). Coordination of Ru(III) ions with POP-bp/bbp significantly changes the electron density at the pyridinic N sites and thus the binding energies of their N 1s electrons. The N 1s XPS profile of POP-bp/bbp was deconvoluted into two peaks centered at 398.84 and 400.47 eV, which are assigned to pyridinic N and imidazolic N, respectively (Figure 1c). After the loading of RuCl_3_, a new N 1s XPS peak was observed at 399.71 eV, corresponding to Ru-bound N species [66]. The binding energy of Ru 3p_3/2_ was 463.35 eV, indicating that the Ru species maintained its original oxidation state of Ru^3+^ in POP-bp/bbpRuCl_3_ [67]. Compared with the reported binding energy of Ru 3p_3/2_ of RuCl_3_ (464.1 eV), the down-shift (0.75 eV) of coordinated Ru^3+^ can be attributed to additional electron density from the strongly electron-donating ligands [68]. High-resolution transmission electron microscopy (HR-TEM) (Figure 1e) did not reveal the presence of Ru nanoparticles. EDX elemental mapping confirmed that the Ru was distributed evenly throughout the POP-bp/bbpRuCl_3_ (Figure 1f). The C, N and Ru contents of the as-synthesized POP-bp/bbpRuCl_3_ were 80.5 wt %, 14.6 wt %, and 4.8 wt %, respectively (Figure S11). The Ru content obtained by EDX element mapping analysis is higher than that calculated by the ICP-OES analysis, which is attributed to the fact that Ru is mainly loaded on the surface of bp/bbp material.

### 3.2. Catalytic β-Alkylation of Secondary Alcohols

We next determined the performance of as-prepared POP-bp/bbpRuCl_3_ as a catalyst for the catalytic acceptorless dehydrogenation coupling of alcohols. The reaction of 1-phenylethanol (mol %) with benzyl alcohol (2a) was conducted in toluene with KOH under a nitrogen atmosphere (Table 1). A standard workup produced 1-([1,1′-biphenyl]-4-yl)ethan-1-one (3aa) in 68% yield with a trace of 1,3-diphenylpropan-1-one (4aa) as determined by high-performance liquid chromatography (HPLC). The screening of different bases (entries 1–5) revealed that CsOH was more selective than KOBu^t^, Cs_2_CO_3_ or NaOH, but that KOH facilitated the highest yield. Lowering the reaction temperature to 100 °C or 120 °C reduced the yield of 3aa significantly, while raising it slightly (140 °C) had a modest impact (entries 6–9). The optimal amount of KOH was 0.5 equivalents (entries 10–13). The yield of 3aa could be increased up to 93% by extending the reaction time to 12 h (entries 15). Interestingly, neither of the individual components of the catalyst (i.e., POP-bp/bbp or RuCl_3_) facilitated more than a very modest amount of product under these conditions (entries 16 and 17).

Substrate scope was investigated with a variety of secondary and primary alcohols under the optimized conditions (Table 2). Good to high yields of β-alkylation were obtained with a variety of primary alcohols as the cross-coupling partner. The reaction of 1-phenylethanol with benzyl alcohols bearing both electron-donating and electron-withdrawing groups at the *para*-position resulted in products 3ab-3af in good yields (73–87%). Substrates bearing *ortho*- and *meta*-methoxy, methyl or chloro also delivered the expected products 3ag-3ak in good yields (79–89%). Likewise, 1-naphthylmethanol and 3,4-methylenedioxybenzyl alcohol reacted with 1a to form 3al and 3am in yields of 88% and 80%, respectively. Heterocyclic 2-thiophenemethanol 2n reacted with 1-phenylethanol less efficiently, giving 3an in 59% yield.

Next, the generality of this catalytic alkylation was investigated with various secondary alcohols (Table 3). The reaction of benzyl alcohol with *para*-substituted 1-phenylethanols bearing either electron withdrawing (-Cl, -Br) or electron-donating (-Me, -OMe) groups afforded the desired β-alkylated secondary alcohols 3ba-3ea in excellent yields (80–89%). No obvious substituent effect was observed. Chloro, methyl and methoxy groups at the *ortho* position of 1-phenylethanol gave the desired secondary alcohols 3fa-3ga in lower yields (53–79%) probably due to steric hindrance. Substrates with methoxy and methyl groups at the *meta*-position gave 3ia and 3ja in good yields (85 and 88%). 1-(Naphthalen-2-yl)ethanol was successfully converted to the coupled product 3ka (90%).

**Figure 2:**
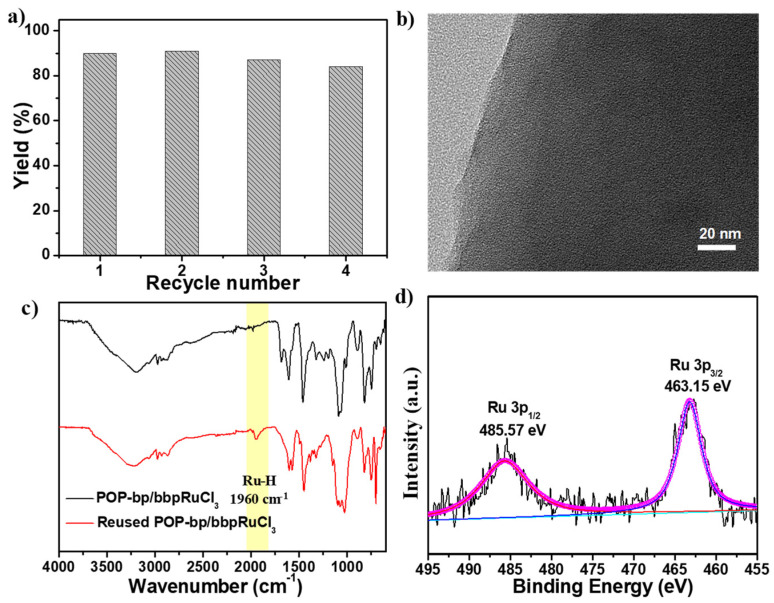
(**a**) Stability of the POP-bp/bbpRuCl_3_ catalyst. (**b**) Transmission electron microscopy (TEM) image of the reused POP-bp/bbpRuCl_3_. (**c**) FT-IR spectra of fresh and reused POP-bp/bbpRuCl_3_. (**d**) Deconvoluted Ru 3p XPS spectrum POP-bp/bbpRuCl_3_.

The recycling of POP-bp/bbpRuCl_3_ was examined under optimized reaction conditions. No significant loss of catalytic activity was observed after four cycles (Figure 2a) and ICP analysis indicated that 96% of Ru remained. Ru nanoparticles were not observed in the subsequent TEM images (Figure 2b). EDX analysis revealed the homogeneous distribution of Ru components throughout the polymer POP-bp/bbp (Figure S12). The FT-IR spectrum of the recovered catalyst did not change noticeably (Figure 2c), except for a new peak at 1960 cm^−1^ which we assign to a Ru-H species [69] generated during the catalytic cycle. XPS measurements indicated that the Cl 2p peak had disappeared in the recovered catalyst indicating ligand substitution (Figure S13). The binding energies of Ru 3p_3/2_ and Ru 3d_5/2_ for the catalyst were 463.15 eV and 281.4 eV, respectively (Figure 2d and Figure S14). The slight decrease in binding energies compared to that of fresh catalyst may be due to the substitution of hydride, which has a greater electron donating ability than chloride [69]. To verify whether the observed catalysis was due to the heterogeneous catalyst POP-bp/bbpRuCl_3_ or due to leached ruthenium species, a reaction was performed between 1a and 2a under standard conditions. The yield of 3aa was 42% accompanied by 7% 4aa after 2h. The reaction was then filtered. No catalytic function was observed in the filtered solution over 24 h and negligible Ru content was detected by ICP. These results indicate that the POP-bp/bbpRuCl_3_ catalyst is stable, which we ascribe to the strong binding of pincer ligand to metal centers.

**Scheme 2:**
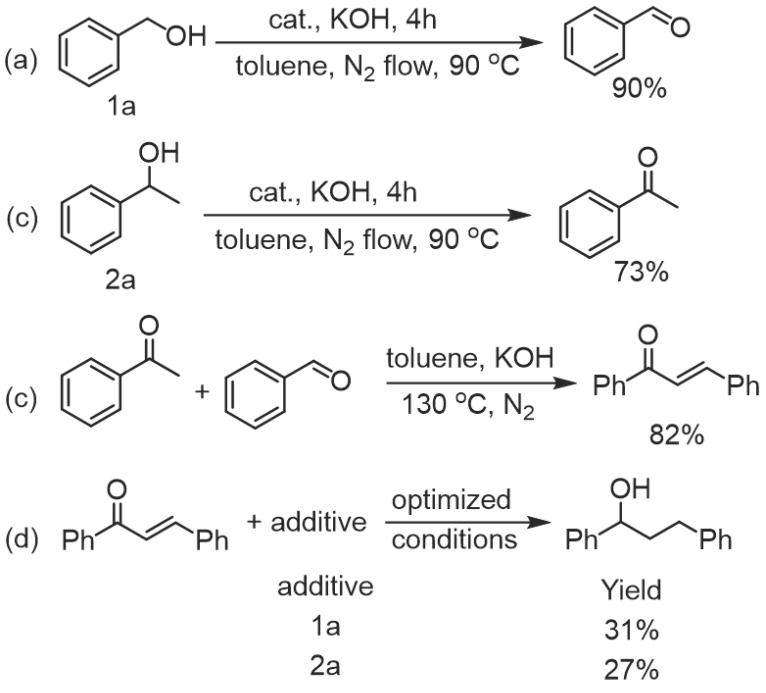
Mechanism elucidation experiments.

A probable mechanism of this reaction was determined by investigating each step individually. The POP-bp/bbpRuCl_3_-catalysed dehydrogenation of phenylmethanol and 1-phenylethanol for 4 h afforded benzaldehyde in 90% yield and acetophenone in 73% yield, respectively (Scheme 2(a,b)). The condensation of benzaldehyde with acetophenone, facilitated by KOH afforded chalcone in good yield (Scheme 2(c)). reduction of chalcone with 1a under the standard reaction conditions achieved 31% of the corresponding secondary alcohol. as shown in Scheme 2, hydrogenation of chalcone with 2a under the same reaction conditions also gave the same reduced product in a similar 27% yield (Scheme 2(d)).

**Scheme 3:**
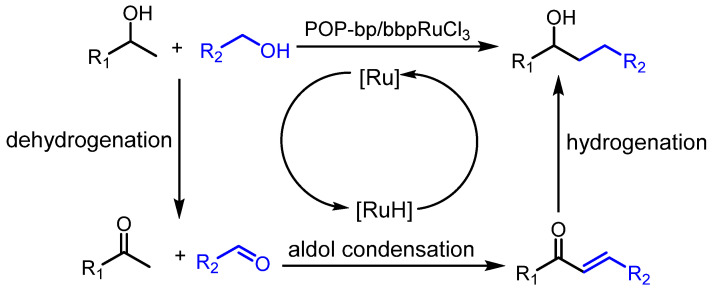
Proposed mechanism.

In light of these results, and of previous literature reports [14,15,16,17], a catalytic mechanism is proposed (Scheme 3). Primary and secondary alcohols are dehydrogenated by the Ru catalyst to form the corresponding aldehyde, ketone and a ruthenium hydride complex. Base-catalysed aldol condensation of the resulting ketone and aldehyde give the α,β-unsaturated ketone intermediate which is reduced by the ruthenium hydride to generate β-alkylated secondary alcohol.

## 4. Conclusions

A novel porous organic polymer incorporating a NNN pincer ligand possessed excellent thermal durability and high surface area. This acceptorless dehydrogenation, cross-coupling catalyst exhibited good activity, broad substrate scope and good recycling ability. We believe this work provides a green, convenient and scalable method for constructing C–C bonds, combining many of the advantages of homogeneous and heterogeneous catalysis.

## Figures and Tables

**Table 1:**
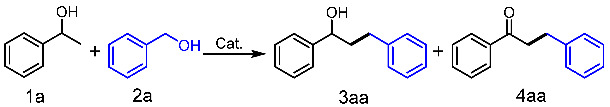
Optimization of the reaction conditions.

Entry ^a^	Catalyst	Base (equiv.)	Temperature (°C)	Time(h)	Yield (%) 3aa	4aa
1	POP-bp/bbpRuCl_3_	KOH/1.0	130	4	68	<5
2	POP-bp/bbpRuCl_3_	KO*^t^*Bu/1.0	130	4	33	trace
3	POP-bp/bbpRuCl_3_	Cs_2_CO_3_/1.0	130	4	32	<5
4	POP-bp/bbpRuCl_3_	NaOH/1.0	130	4	11	trace
5	POP-bp/bbpRuCl_3_	CsOH/1.0	130	4	45	<5
6	POP-bp/bbpRuCl_3_	KOH/1.0	100	4	7	25
7	POP-bp/bbpRuCl_3_	KOH/1.0	110	4	20	14
8	POP-bp/bbpRuCl_3_	KOH/1.0	120	4	49	20
9	POP-bp/bbpRuCl_3_	KOH/1.0	140	4	70	trace
10	POP-bp/bbpRuCl_3_	KOH/0.3	130	4	60	12
11	POP-bp/bbpRuCl_3_	KOH/0.5	130	4	76	8
12	POP-bp/bbpRuCl_3_	KOH/0.7	130	4	75	6
13	POP-bp/bbpRuCl_3_	KOH/1.5	130	4	69	<5
14	POP-bp/bbpRuCl_3_	KOH/1.0	130	8	82	<5
15	POP-bp/bbpRuCl_3_	KOH/0.5	130	12	93	trace
16	POP-bp/bbp	KOH/0.5	130	12	0	trace
17	RuCl_3_	KOH/0.5	130	12	18	trace

^a^ 130 Reaction conditions: 1a (1 mmol), 2a (1.2 mmol), cat. (20 mg, 0.6 mol% Ru), base (eq.), toluene (2 mL) in a 15 mL sealed tube, at 130 °C for 4 h, high-performance liquid chromatography (HPLC) yield using biphenyl as the internal standard.

**Table 2:**
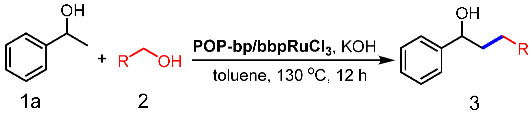
β-Alkylation of 1-phenylethanol with various primary alcohols.

Entry ^a^	Primary Alcohol		Product	Yield (%)	
1	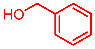	2a	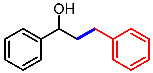	3aa	88
2	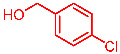	2b	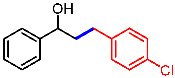	3ab	85
3	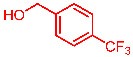	2c	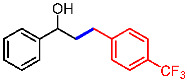	3ac	73
4	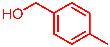	2d	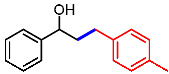	3ad	85
5	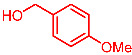	2e	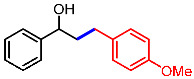	3ae	87
6	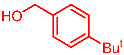	2f	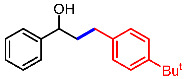	3af	87
7	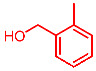	2g	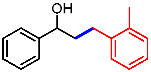	3ag	89
8	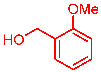	2h	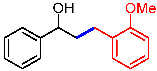	3ah	79
9	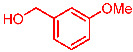	2i	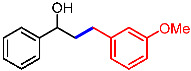	3ai	86
10	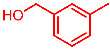	2j	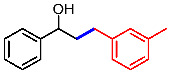	3aj	81
11	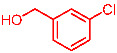	2k	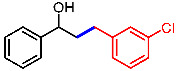	3ak	85
12	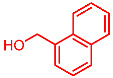	2l	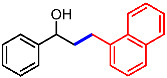	3al	88
13	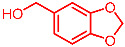	2m	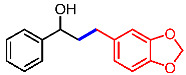	3am	80
14	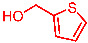	2n	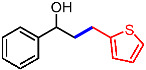	3an	59

^a^ Reaction condition: 1a (1 mmol), primary alcohol (1.2 mmol), POP-bp/bbpRuCl_3_ (20 mg, 0.6 mol % Ru), KOH (0.5 equiv.), toluene (2 mL), 130 °C, 12 h, isolated yields.

**Table 3:**
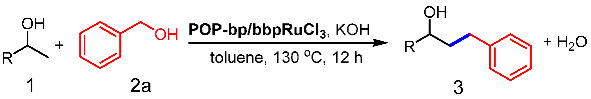
β-Alkylation of various secondary alcohols with benzyl alcohol.

Entry ^a^	Secondary Alcohol		Product	Yield (%)	
1	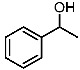	1a	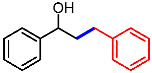	3aa	88
2	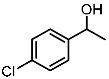	1b	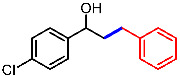	3ba	80
3	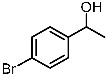	1c	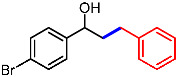	3ca	89
4	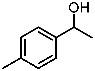	1d	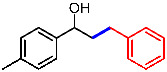	3da	81
5	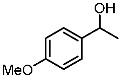	1e	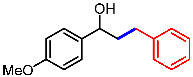	3ea	88
6	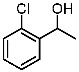	1f	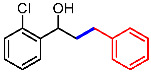	3fa	53
7	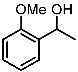	1g	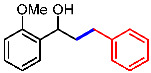	3ga	76
8	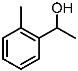	1h	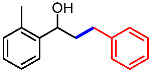	3ha	79
9	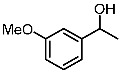	1i	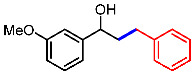	3ia	88
10	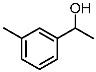	1j	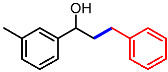	3ja	85
11	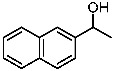	1k	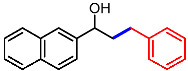	3ka	90

^a^ Reaction condition: secondary alcohol (1 mmol), 2a (1.2 mmol), POP-bp/bbpRuCl_3_ (20 mg, 0.6 mol%Ru), KOH (0.5 equiv.), toluene (2 mL), 130 °C, 12 h, isolated yields.

## Data Availability

The data presented in this study are available on request from the corresponding author.

## Supplementary Materials

The following supporting information can be downloaded at: https://www.mdpi.com/article/10.3390/polym14020231/s1, Figure S1: TGA curve. Figure S2: ^13^C CP-MAS NMR spectrum. Figure S3: SEM image. Figure S4: TEM image. Figure S5: EDS elemental mapping. Figure S6: FT-IR spectrum. Figure S7: Pore-size distribution. Figure S8: PXRD patterns. Figure S9: XPS survey scan spectrum. Figure S10: XPS spectra. Figures S11 and S12: EDX spectroscopy elemental mapping. Figures S13 and S14: XPS survey scan spectra. Figures S15–S38: NMR data of products. References [12,31,70,71,72,73] are cited in the supplementary materials.
