# Supplementary material for: Construction of a (NNN)Ru-Incorporated Porous Organic Polymer with High Catalytic Activity for β-Alkylation of Secondary Alcohols with Primary Alcohols
Source: Polymers (Basel). 2022 Jan 7;14(2):231. doi: 10.3390/polym14020231 (PMC8780954; doi:10.3390/polym14020231)

Construction of a (NNN)Ru-incorporated porous organic polymer with high catalytic activity for β-alkylation of secondary alcohols with primary alcohols

Yao Cui [1], Jixian Wang [1], Lei Yu [2], Ying Xu [2], David J. Young [3], Hai-Yan Li [2] Hong-Xi Li*,[1]

[1] College of Chemistry, Chemical Engineering and Materials Science, Soochow University, Suzhou 215123, People's Republic of China

[2] Analysis and Testing Centre, Soochow University, Suzhou 215123, People's Republic of China

[3] College of Engineering, Information Technology and Environment, Charles Darwin University, Darwin, NT, 0909, Australia

Corresponding Authors:

*E-mail: lihx@suda.edu.cn.

Table of Contents

Figure S1. TGA plot of POP-bp/bbp..S3

Figure S2. ^{13}C CP-MAS NMR spectrum of POP-bp/bbp.. ....S3

Figure S3. SEM image of POP-bp/bbp. S4

Figure S4. TEM image of POP-bp/bbp. S4

Figure S5. EDS elemental mapping of POP-bp/bbp. S4

Figure S6. FT-IR spectra of bp, bbp, and POP-bp/bbp. S5

Figure S7. Pore-size distribution of POP-bp/bbp and POP-bp/bbpRuCl_3 analyzed by NLDFT. ...S5

Figure S8. PXRD patterns of POP-bp/bbp and POP-bp/bbpRuCl_3..S6

Figure S9. XPS survey scan spectra of POP-bp/bbpRuCl_3. ....S6

Figure S10. Ru 3d XPS spectra of POP-bp/bbpRuCl_3. ....S7

Figure S11. EDX spectroscopy elemental mapping of the as-synthesized POP-bp/bbpRuCl_3.....S7

Figure S12. EDX spectroscopy elemental mapping of the reused POP-bp/bbpRuCl_3. ....S7

Figure S13. XPS survey scan spectrum of the reused POP-bp/bbpRuCl_3. ....S8

Figure S14. Ru 3d XPS spectra of the reused POP-bp/bbpRuCl_3. ....S8

^{1}H and ^{13}C NMR data of products...S9

References S17

NMR spectra...S18

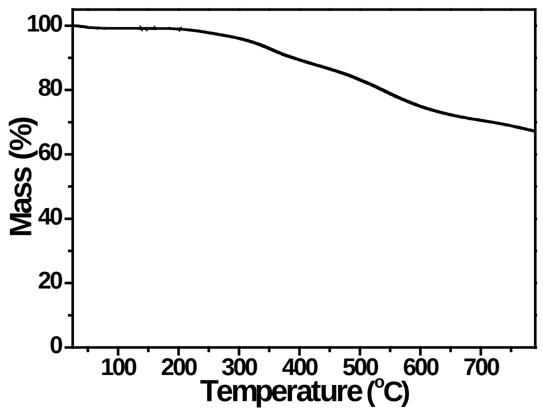

Figure S1. TGA plot of POP-bp/bbp.

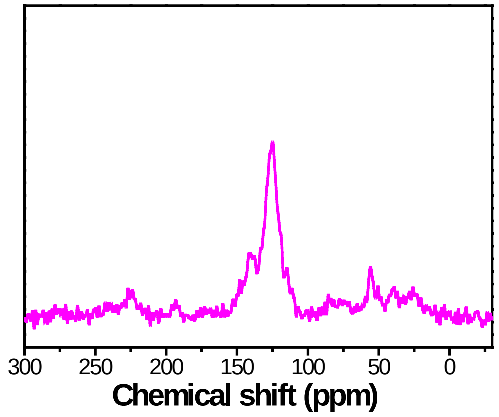

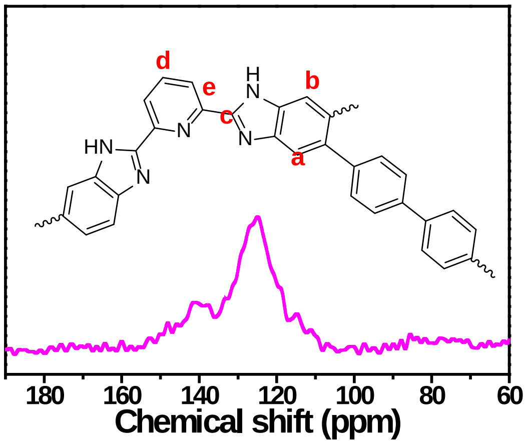

Figure S2. ^{13}C CP-MAS NMR spectrum of POP-bp/bbp.

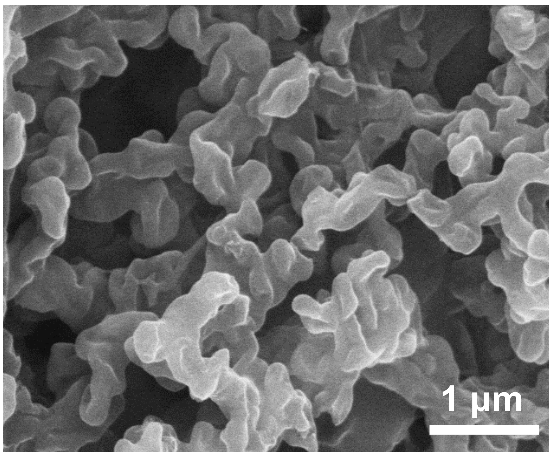

Figure S3. SEM image of POP-bp/bbp.

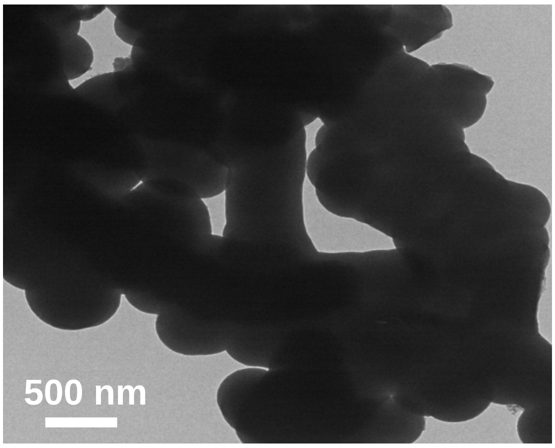

Figure S4. TEM image of POP-bp/bbp.

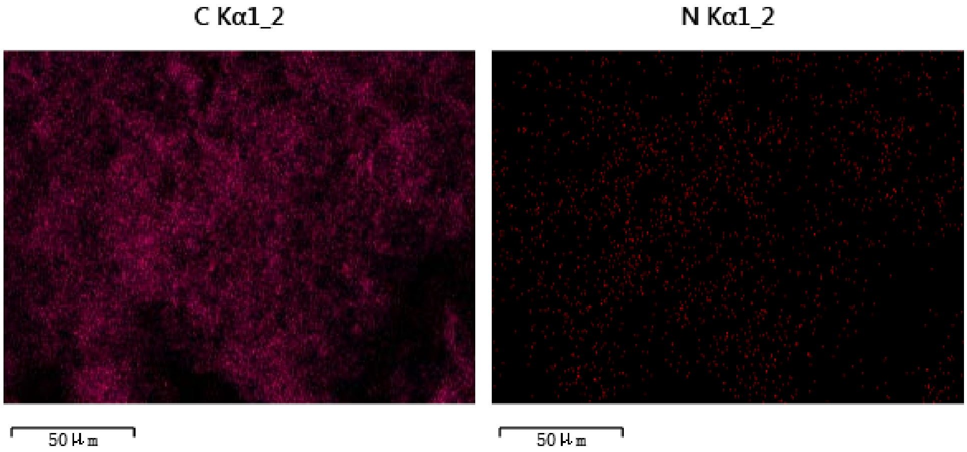

Figure S5. EDS elemental mapping of POP-bp/bbp.

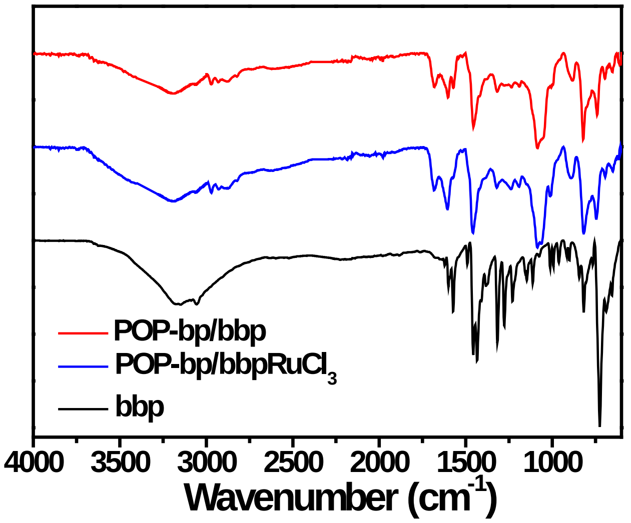

Figure S6. FT-IR spectra of bp, bbp, and POP-bp/bbp.

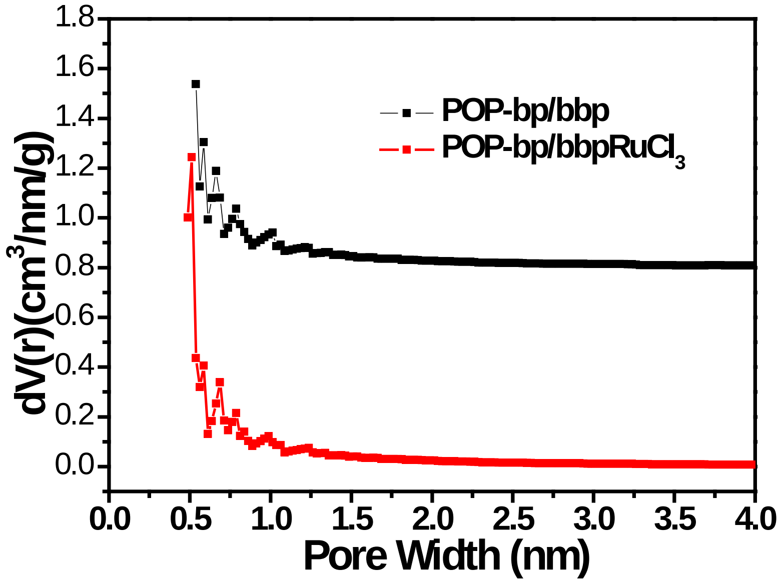

Figure S7. Pore-size distribution of POP-bp/bbp and POP-bp/bbp$RuCl_3$ analyzed by NLDFT.

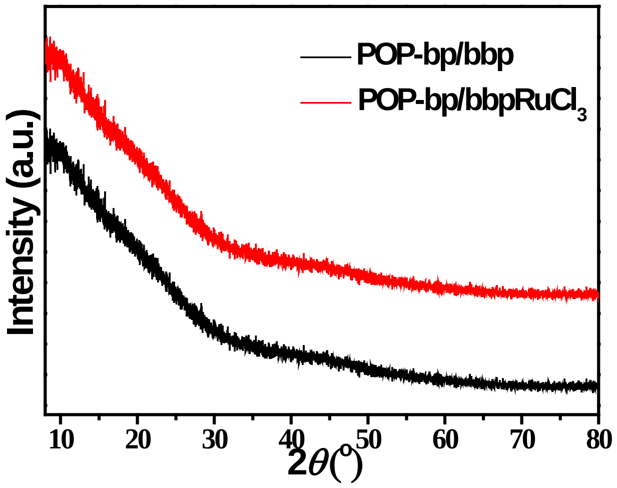

Figure S8. PXRD patterns of POP-bp/bbp and POP-bp/bbpRuCl_3.

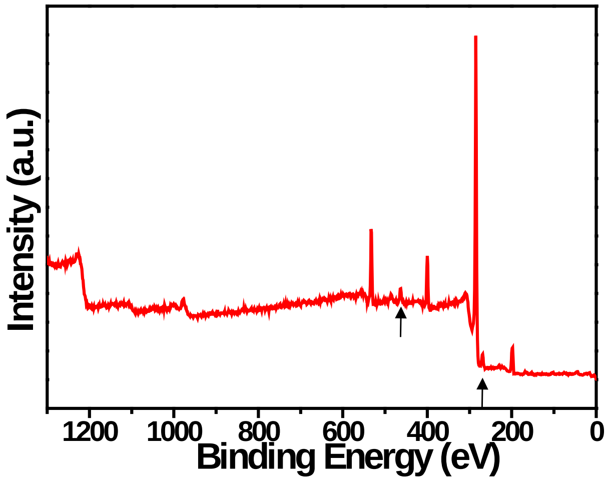

Figure S9. XPS survey scan spectra of POP-bp/bbpRuCl_3.

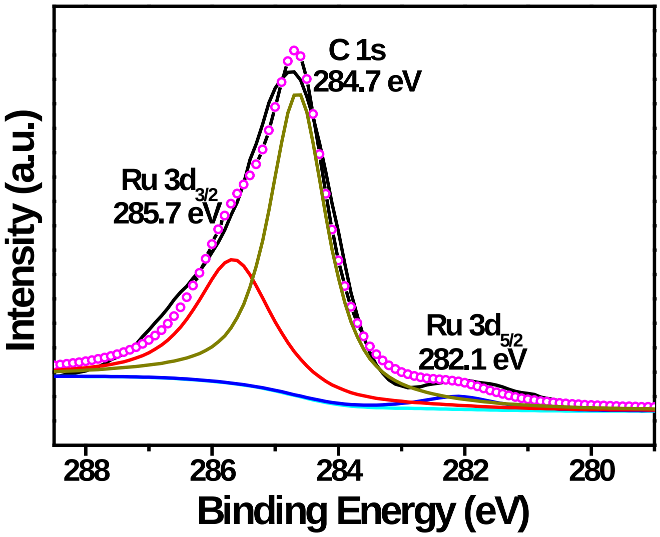

Figure S10. Ru 3d XPS spectra of POP-bp/bbpRuCl$_3$.

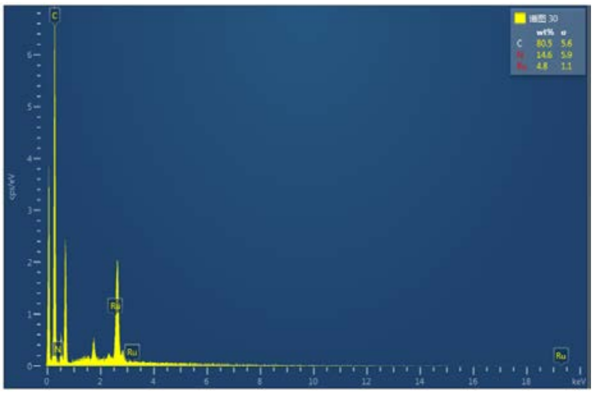

Figure S11. EDX spectroscopy elemental mapping of the as-synthesized POP-bp/bbpRuCl$_3$.

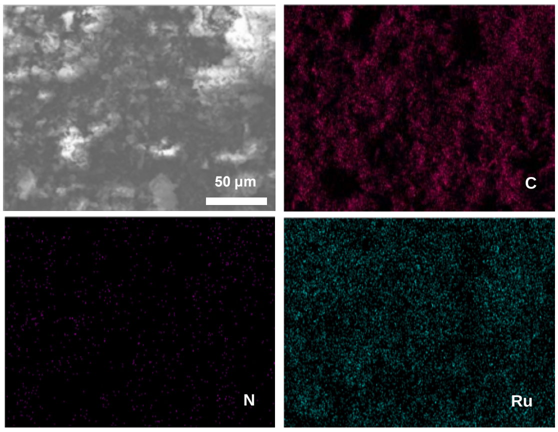

Figure S12. EDX spectroscopy elemental mapping of the reused POP-bp/bbpRuCl$_3$.

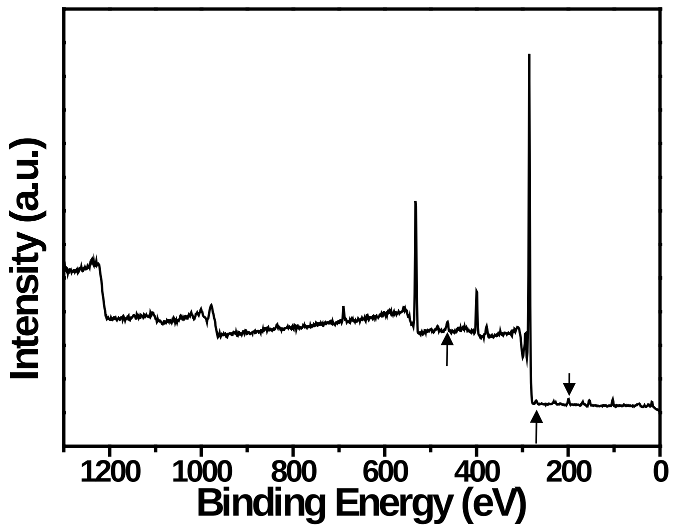

Figure S13. XPS survey scan spectrum of the reused POP-bp/bbpRuCl_3.

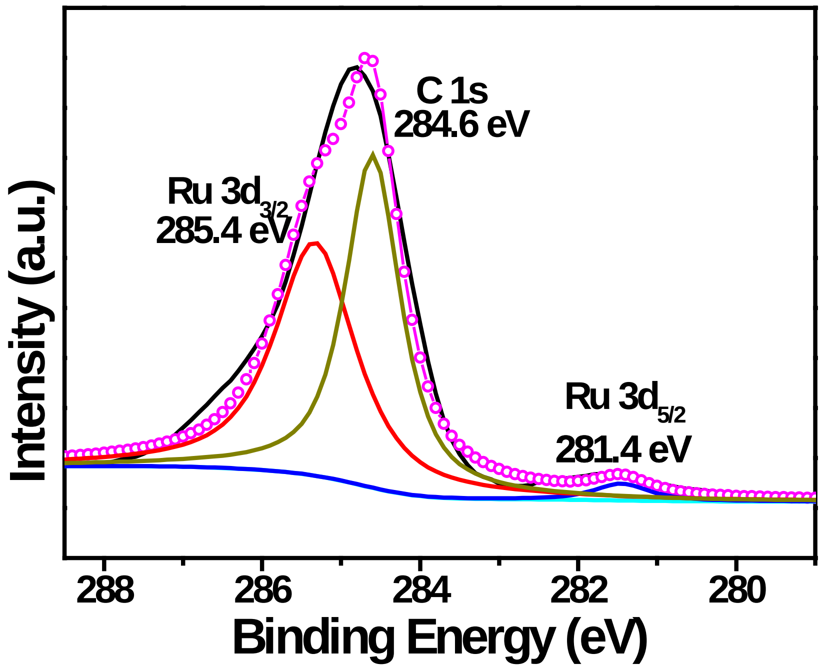

Figure S14. Ru 3d XPS spectra of the reused POP-bp/bbpRuCl_3.

Analytical data of the products

1,3-diphenylpropan-1-ol (3aa) [S1].

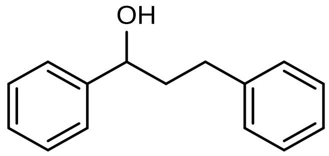

186 mg, 88% yield. Colorless oil. ^{1}H NMR (400 MHz, $CDCl_3$) δ 7.34–7.26 (m, 4H), 7.26–7.20 (m, 3H), 7.15 (t, J = 6.2 Hz, 3H), 4.59 (dd, J = 7.4, 5.7 Hz, 1H), 2.65 (m, 2H), 2.23 (s, 1H), 2.14–1.86 (m, 2H). ^{13}C NMR (100 MHz, $CDCl_3$) δ 144.7, 141.9, 128.54, 128.51, 128.45, 127.7, 126.0, 125.9, 73.9, 40.5, 32.1.

3-(4-chlorophenyl)-1-phenylpropan-1-ol (3ab) [S1].

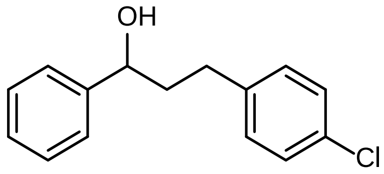

209 mg, 85% yield. White solid. ^{1}H NMR (400 MHz, $CDCl_3$) δ 7.34 (q, J = 6.2 Hz, 4H), 7.25 (dd, J = 16.2, 9.5 Hz, 3H), 7.11 (d, J = 8.3 Hz, 2H), 4.65 (t, J = 4.7 Hz, 1H), 2.82–2.54 (m, 2H), 2.19–1.88 (m, 3H). ^{13}C NMR (100 MHz, $CDCl_3$) δ 144.6, 140.4, 131.7, 130.0, 128.7, 128.6, 127.9, 126.1, 73.9, 40.5, 31.5.

1-phenyl-3-(4-(trifluoromethyl)phenyl)propan-1-ol (3ac) [S2].

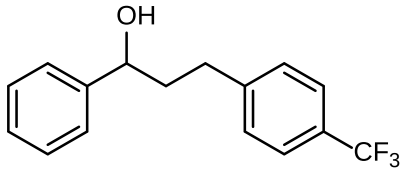

204 mg, 73% yield. Colorless oil. ^{1}H NMR (400 MHz, $CDCl_3$) δ 7.49 (d, J = 8.1 Hz, 2H), 7.31 (s, 1H), 7.28 (dd, J = 8.6, 2.1 Hz, 3H), 7.23 (t, J = 5.8 Hz, 3H), 4.59 (dd, J = 7.7, 5.5 Hz, 1H), 2.83–2.56 (m, 2H), 2.48 (s, 1H), 2.17–1.85 (m, 2H). ^{13}C NMR (100 MHz, $CDCl_3$) δ 146.1, 144.4, 128.8, 128.7, 127.9, 126.0, 125.4, 125.4, 125.3, 125.3, 73.7, 40.1, 31.9. ^{19}F NMR (377 MHz, $CDCl_3$) δ -62.15.

1-phenyl-3-(p-tolyl)propan-1-ol (3ad) [S1].

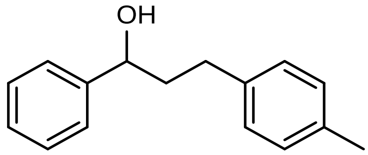

192 mg, 85% yield. White solid. ^{1}H NMR (400 MHz, $CDCl_3$) δ 7.29 (s, 4H), 7.24 (d, J = 5.7 Hz, 1H), 7.05 (s, 4H), 4.66–4.53 (m, 1H), 2.79–2.49 (m, 2H), 2.29 (s, 3H), 2.21 (s, 1H), 2.01 (m, 2H). ^{13}C NMR (100 MHz, $CDCl_3$) δ 144.7, 138.8, 135.3, 129.1, 128.5, 128.4, 127.6, 126.0, 73.9, 40.6, 31.6, 21.1.

3-(4-methoxyphenyl)-1-phenylpropan-1-ol (3ae) [S1].

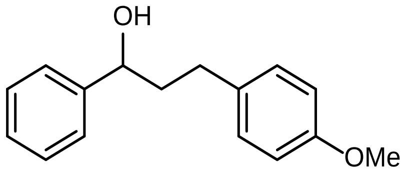

210 mg, 87% yield. Colorless oil. ^{1}H NMR (400 MHz, $CDCl_3$) δ 7.34 (d, J = 2.3 Hz, 4H), 7.31–7.25 (m, 1H), 7.10 (dd, J = 8.1, 3.8 Hz, 2H), 6.83 (dd, J = 8.1, 5.8 Hz, 2H), 4.74–4.61 (m, 1H), 3.78 (s, 3H), 2.78–2.54 (m, 2H), 2.23–1.83 (m, 3H). ^{13}C NMR (100 MHz, $CDCl_3$) δ 157.9, 144.8, 134.0, 129.5, 128.6, 127.7, 126.1, 114.0, 74.0, 55.4, 40.9, 31.3.

3-(4-(tert-butyl)phenyl)-1-phenylpropan-1-ol (3af) [S3].

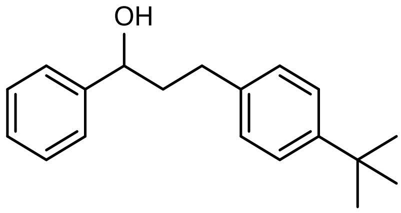

233 mg, 87% yield. Colorless oil. ^{1}H NMR (400 MHz, $CDCl_3$) δ 7.33–7.25 (m, 6H), 7.25–7.19 (m, 1H), 7.09 (d, J = 8.3 Hz, 2H), 4.60 (t, J = 6.5 Hz, 1H), 2.78–2.48 (m, 2H), 2.27 (s, 1H), 2.03 (m, 2H), 1.29 (s, 9H). ^{13}C NMR (100 MHz, $CDCl_3$) δ 148.7, 144.7, 138.8, 128.5, 128.2, 127.6, 126.1, 125.3, 73.9, 40.5, 34.4, 31.5, 31.5.

1-phenyl-3-(o-tolyl)propan-1-ol (3ag) [S1].

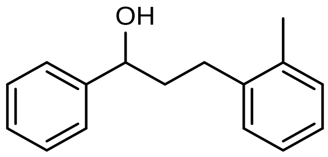

201 mg, 89% yield. Colorless oil. ^{1}H NMR (400 MHz, $CDCl_3$) δ 7.27 (d, J = 4.4 Hz, 4H), 7.23–7.19 (m, 1H), 7.07 (dd, J = 5.9, 3.2 Hz, 4H), 4.60 (dd, J = 7.6, 5.5 Hz, 1H), 2.72–2.63 (m, 1H), 2.58–2.49 (m, 1H), 2.41 (s, 1H), 2.20 (s, 3H), 2.02–1.87 (m, 2H). ^{13}C NMR (100 MHz, $CDCl_3$) δ 144.6, 140.1, 136.0, 130.2, 128.8, 128.5, 127.6,

125.99, 125.98, 74.1, 39.2, 29.4, 19.2.

3-(2-methoxyphenyl)-1-phenylpropan-1-ol (3ah) [S4].

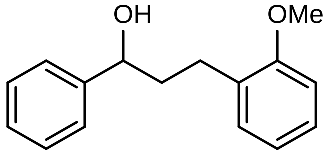

191 mg, 79% yield. Colorless oil. ^{1}H NMR (400 MHz, $CDCl_3$) δ 7.28 (dd, J = 9.1, 5.2 Hz, 4H), 7.22–7.08 (m, 3H), 6.89–6.77 (m, 2H), 4.56 (dd, J = 8.3, 5.0 Hz, 1H), 3.74 (s, 3H), 2.70 (m, 2H), 2.65 (s, 1H), 2.07–1.91 (m, 2H). ^{13}C NMR (100 MHz, $CDCl_3$) δ 157.4, 144.7, 130.1, 130.1, 128.3, 127.3, 127.2, 126.0, 120.7, 110.4, 73.6, 55.3, 39.3, 26.5.

3-(3-methoxyphenyl)-1-phenylpropan-1-ol (3ai) [S1].

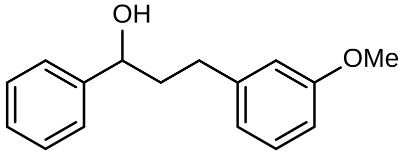

208 mg, 86% yield. Colorless oil. ^{1}H NMR (400 MHz, $CDCl_3$) δ 7.38–7.19 (m, 5H), 7.20–7.12 (m, 1H), 6.72 (dd, J = 19.4, 7.1 Hz, 3H), 4.65–4.52 (m, 1H), 3.72 (s, 3H), 2.78–2.50 (m, 2H), 2.35 (s, 1H), 2.16–1.89 (m, 2H). ^{13}C NMR (100 MHz, $CDCl_3$) δ 159.7, 144.7, 143.5, 129.4, 128.5, 127.60, 126.0, 120.9, 114.3, 111.2, 73.8, 55.1, 40.4, 32.1.

1-phenyl-3-(m-tolyl)propan-1-ol (3aj) [S1].

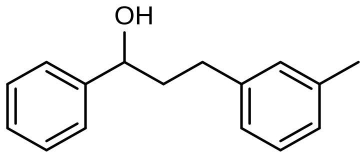

183 mg, 81% yield. Colorless oil. ^{1}H NMR (400 MHz, $CDCl_3$) δ 7.28 (s, 4H), 7.24 (d, J = 5.8 Hz, 1H), 7.13 (t, J = 7.7 Hz, 1H), 7.02–6.87 (m, 3H), 4.71–4.45 (m, 1H), 2.72–2.51 (m, 2H), 2.29 (s, 4H), 2.00 (m, 2H). ^{13}C NMR (100 MHz, $CDCl_3$) δ 144.7, 141.8, 137.9, 129.3, 128.5, 128.3, 127.6, 126.6, 126.0, 125.5, 73.9, 40.5, 32.0, 21.5.

3-(3-chlorophenyl)-1-phenylpropan-1-ol (3ak) [S1].

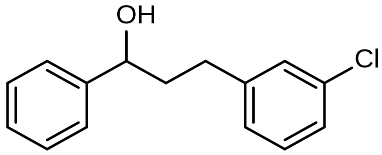

209 mg, 85% yield. Colorless oil. ^{1}H NMR (400 MHz, $CDCl_3$) δ 7.33–7.30 (m, 1H), 7.29 (s, 1H), 7.26 (dd, J = 11.3, 4.8 Hz, 2H), 7.17–7.10 (m, 3H), 7.00 (dt, J = 6.5, 1.7 Hz, 1H), 4.57 (t, J = 6.5 Hz, 1H), 2.60 (m, 2H), 2.41 (s, 1H), 2.10–1.86 (m, 2H). ^{13}C NMR (100 MHz, $CDCl_3$) δ 144.4, 144.0, 134.1, 129.7, 128.6, 128.6, 127.8, 126.7, 126.1, 126.0, 73.7, 40.2, 31.7.

3-(naphthalen-1-yl)-1-phenylpropan-1-ol (3al) [S1].

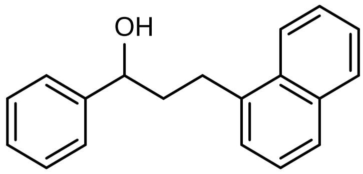

230 mg, 88% yield. White solid. ^{1}H NMR (400 MHz, $CDCl_3$) δ 7.97–7.88 (m, 1H), 7.80 (dd, J = 7.5, 2.0 Hz, 1H), 7.66 (d, J = 8.1 Hz, 1H), 7.47–7.39 (m, 2H), 7.37–7.26 (m, 6H), 7.26–7.19 (m, 1H), 4.69 (dd, J = 7.2, 5.6 Hz, 1H), 3.18 (m, 1H), 3.04 (m, 1H), 2.24–2.04 (m, 3H). ^{13}C NMR (100 MHz, $CDCl_3$) δ 144.6, 138.1, 134.0, 131.9, 128.8, 128.6, 127.7, 126.8, 126.0, 125.9, 125.6, 125.5, 123.9, 74.2, 39.9, 29.2.

3-(benzo[d][1,3]dioxol-5-yl)-1-phenylpropan-1-ol (3am) [S1].

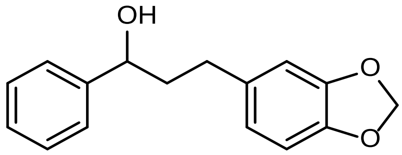

205mg, 80% yield. White solid. ^{1}H NMR (400 MHz, $CDCl_3$) δ 7.34–7.27 (m, 4H), 7.26–7.20 (m, 1H), 7.07 (dd, J = 5.1, 1.1 Hz, 1H), 6.88 (dd, J = 5.1, 3.4 Hz, 1H), 6.76 (dd, J = 3.4, 0.9 Hz, 1H), 4.63 (dd, J = 7.8, 5.4 Hz, 1H), 2.87 (m, 2H), 2.32 (s, 1H), 2.06 (m, 2H). ^{13}C NMR (100 MHz, $CDCl_3$) δ 144.7, 144.4, 128.6, 127.7, 126.8, 126.0, 124.4, 123.1, 73.5, 40.7, 26.2.

1-phenyl-3-(thiophen-2-yl)propan-1-ol (3an) [S2].

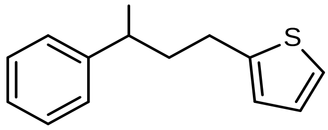

129 mg, 59% yield. Colorless oil. ^{1}H NMR (400 MHz, $CDCl_3$) δ 7.37–7.28 (m, 4H), 7.28–7.22 (m, 1H), 6.67 (dd, J = 16.4, 4.7 Hz, 2H), 6.60 (dd, J = 7.9, 1.6 Hz, 1H), 5.86 (s, 2H), 4.61 (dd, J = 7.8, 5.4 Hz, 1H), 2.70–2.48 (m, 2H), 2.16 (s, 1H), 2.08–

1.85 (m, 2H). ^{13}C NMR (100 MHz, $CDCl_3$) δ 147.6, 145.7, 144.7, 135.7, 128.6, 127.7, 126.0, 121.2, 109.0, 108.2, 100.8, 73.7, 40.8, 31.8.

1-(4-chlorophenyl)-3-phenylpropan-1-ol (3ba) [S1].

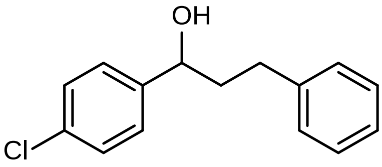

197 mg, 80% yield. Colorless oil. ^{1}H NMR (400 MHz, $CDCl_3$) δ 7.31 (d, J = 8.3 Hz, 2H), 7.28 (t, J = 7.7 Hz, 4H), 7.18 (t, J = 8.6 Hz, 3H), 4.69–4.62 (m, 1H), 2.77–2.61 (m, 2H), 2.09 (m, 1H), 1.98 (m, 1H), 1.81 (s, 1H). ^{13}C NMR (100 MHz, $CDCl_3$) δ 143.2, 141.6, 133.4, 128.8, 128.6, 128.5, 127.4, 126.1, 73.3, 40.6, 32.1.

1-(4-bromophenyl)-3-phenylpropan-1-ol (3ca) [S1].

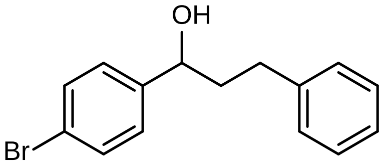

259 mg, 895% yield. Colorless oil. ^{1}H NMR (400 MHz, $CDCl_3$) δ 7.47 (d, J = 8.3 Hz, 2H), 7.28 (t, J = 7.5 Hz, 2H), 7.22 (d, J = 8.3 Hz, 2H), 7.18 (t, J = 8.3 Hz, 3H), 4.65 (dd, J = 7.6, 5.5 Hz, 1H), 2.78–2.61 (m, 2H), 2.16–2.05 (m, 1H), 2.03–1.93 (m, 1H), 1.77 (s, 1H). ^{13}C NMR (100 MHz, $CDCl_3$) δ 142.7, 140.6, 130.7, 127.7, 127.6, 127.6, 126.8, 125.1, 125.1, 120.5, 72.3, 39.6, 31.0.

3-phenyl-1-(p-tolyl)propan-1-ol (3da) [S1].

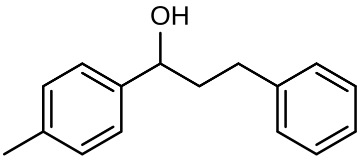

183 mg, 81% yield. Colorless oil. ^{1}H NMR (400 MHz, $CDCl_3$) δ 7.27–7.21 (m, 2H), 7.19 (s, 1H), 7.18–7.08 (m, 6H), 4.63–4.51 (m, 1H), 2.76–2.54 (m, 2H), 2.31 (s, 3H), 2.19 (s, 1H), 2.12–2.01 (m, 1H), 2.01–1.90 (m, 1H). ^{13}C NMR (100 MHz, $CDCl_3$) δ 142.0, 141.7, 137.3, 129.2, 128.5, 128.4, 126.0, 125.9, 73.7, 40.4, 32.1, 21.2.

1-(4-methoxyphenyl)-3-phenylpropan-1-ol (3ea) [S4].

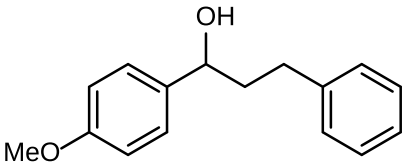

213 mg, 88% yield. White solid. ^{1}H NMR (400 MHz, $CDCl_3$) δ 7.29–7.21 (m, 3H), 7.20 (d, J = 1.9 Hz, 1H), 7.15 (dd, J = 7.1, 5.3 Hz, 3H), 6.90–6.77 (m, 2H), 4.61–4.50 (m, 1H), 3.74 (s, 3H), 2.80–2.50 (m, 2H), 2.29 (s, 1H), 2.14–2.02 (m, 1H), 1.95 (m, 1H). ^{13}C NMR (100 MHz, $CDCl_3$) δ 159.1, 141.9, 136.8, 128.5, 128.4, 127.3, 125.9, 113.9, 73.4, 55.3, 40.4, 32.1.

1-(2-chlorophenyl)-3-phenylpropan-1-ol (3fa) [S5].

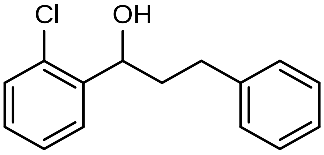

130 mg, 53% yield. Colorless oil. ^{1}H NMR (400 MHz, $CDCl_3$) δ 7.57 (dd, J = 7.7, 1.6 Hz, 1H), 7.34–7.30 (m, 1H), 7.29–7.25 (m, 3H), 7.23–7.19 (m, 3H), 7.17 (d, J = 7.3 Hz, 2H), 5.13 (dd, J = 8.4, 4.1 Hz, 1H), 2.93–2.81 (m, 1H), 2.74 (m, 1H), 2.15–1.99 (m, 2H), 1.85 (s, 1H). ^{13}C NMR (100 MHz, $CDCl_3$) δ 142.1, 141.8, 132.0, 129.6, 129.2, 128.6, 128.5, 128.4, 127.3, 127.2, 126.0, 125.4, 70.4, 39.1, 32.3.

1-(2-methoxyphenyl)-3-phenylpropan-1-ol (3ga) [S1].

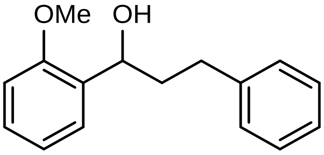

184 mg, 76% yield. Colorless oil. ^{1}H NMR (400 MHz, $CDCl_3$) δ 7.45 (dd, J = 7.5, 1.4 Hz, 1H), 7.42–7.37 (m, 2H), 7.37–7.22 (m, 4H), 7.07 (t, J = 7.3 Hz, 1H), 6.96 (d, J = 8.2 Hz, 1H), 5.03 (s, 1H), 3.89 (s, 3H), 3.03 (s, 1H), 3.00–2.90 (m, 1H), 2.81 (m, 1H), 2.32–2.14 (m, 2H). ^{13}C NMR (100 MHz, $CDCl_3$) δ 156.5, 142.2, 132.4, 128.5, 128.3, 126.9, 125.7, 120.7, 110.5, 70.1, 55.2, 38.8, 32.3.

3-phenyl-1-(o-tolyl)propan-1-ol (3ha) [S1].

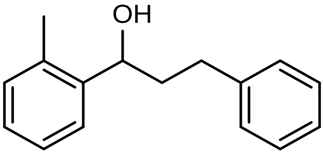

179 mg, 79% yield. Colorless oil. ^{1}H NMR (400 MHz, $CDCl_3$) δ 7.43 (d, J = 7.5 Hz, 1H), 7.28–7.21 (m, 2H), 7.20–7.09 (m, 5H), 7.07 (d, J = 7.2 Hz, 1H), 4.83 (dd, J = 8.0, 4.5 Hz, 1H), 2.79 (m, 1H), 2.67 (m, 1H), 2.17 (s, 3H), 2.10 (s, 1H), 2.05–1.87 (m, 2H). ^{13}C NMR (100 MHz, $CDCl_3$) δ 142.8, 141.9, 134.5, 130.5, 128.5, 128.4, 127.2, 126.3, 125.9, 125.2, 69.9, 39.5, 32.3, 18.9.

1-(3-methoxyphenyl)-3-phenylpropan-1-ol (3ia) [S6].

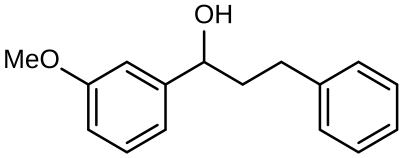

213 mg, 88% yield. Colorless oil. ^{1}H NMR (400 MHz, $CDCl_3$) δ 7.23 (dd, J = 14.1, 7.0 Hz, 3H), 7.16 (t, J = 9.4 Hz, 3H), 6.87 (d, J = 2.0 Hz, 2H), 6.77 (dd, J = 8.1, 1.8 Hz, 1H), 4.57 (dd, J = 7.1, 5.9 Hz, 1H), 3.73 (s, 3H), 2.75–2.53 (m, 2H), 2.42 (s, 1H), 2.14–1.88 (m, 2H). ^{13}C NMR (100 MHz, $CDCl_3$) δ 159.8, 146.4, 141.9, 129.5, 128.5, 128.4, 125.9, 118.3, 113.0, 111.5, 73.7, 55.2, 40.4, 32.1.

3-phenyl-1-(m-tolyl)propan-1-ol (3ja) [S1].

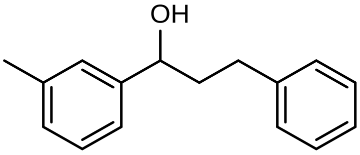

192 mg, 85% yield. Colorless oil. ^{1}H NMR (400 MHz, $CDCl_3$) δ 7.29–7.20 (m, 2H), 7.19 (d, J = 7.5 Hz, 1H), 7.14 (dd, J = 8.4, 4.0 Hz, 3H), 7.12–7.00 (m, 3H), 4.54 (m, 1H), 2.77–2.54 (m, 2H), 2.33 (s, 1H), 2.30 (s, 3H), 2.01 (m, 2H). ^{13}C NMR (100 MHz, $CDCl_3$) δ 144.6, 141.9, 138.1, 128.5, 128.4, 128.4, 126.7, 125.9, 123.1, 73.8, 40.4, 32.1, 21.5.

1-(naphthalen-2-yl)-3-phenylpropan-1-ol (3ka) [S1].

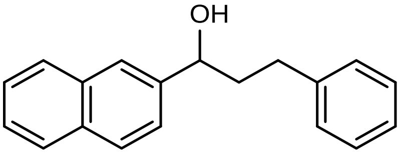

236 mg, 90% yield. White solid. ^{1}H NMR (400 MHz, $CDCl_3$) δ 7.80–7.68 (m, 3H), 7.65 (s, 1H), 7.44–7.33 (m, 3H), 7.22 (t, J = 7.3 Hz, 2H), 7.17–7.09 (m, 3H), 4.69 (dd, J = 7.4, 5.7 Hz, 1H), 2.74 – 2.53 (m, 2H), 2.49 (s, 1H), 2.20–1.93 (m, 2H). ^{13}C NMR (100 MHz, $CDCl_3$) δ 141.9, 141.8, 133.3, 133.0, 128.5, 128.5, 128.4, 128.0, 127.8,

126.2, 125.9, 125.9, 124.7, 124.1, 73.9, 40.3, 32.1.

References

S1 Liu, T.T.; Wang, L.D.; Wu, K.K.; Yu, Z.K. Manganese-catalyzed β-alkylation of secondary alcohols with primary alcohols under phosphine-free conditions, *ACS Catal.* **2018**, *8*, 7201−7207.

S2 Genç, S.; Arslan, B.; Gülcemal, S.; Günnaz, S.; Çetinkaya, B.; Gülcemal, D. Iridium(I)-catalyzed C–C and C–N bond formation reactions via the borrowing hydrogen strategy, *J. Org. Chem.* **2019**, *84*, 6286−6297.

S3 Allen, L.J.; Crabtree, R.H. Green alcohol couplings without transition metal catalysts: base-mediated β-alkylation of alcohols in aerobic conditions, *Green Chem.* **2010**, *12*, 1362–1364.

S4 Wang, Q.F.; Wu, K.K.; Yu, Z.K. Ruthenium(III)-catalyzed β-alkylation of secondary alcohols with primary alcohols, *Organometallics* **2016**, *35*, 1251−1256.

S5 Roy, B.C.; Debnath, S.; Chakrabarti, K.; Paul, B.; Maji, M.; Kundu, S. *ortho*-Amino group functionalized 2,2′-bipyridine based Ru(II) complex catalysed alkylation of secondary alcohols, nitriles and amines using alcohols, *Org. Chem. Front.* **2018**, *5*, 1008–1018.

S6 Zhang, M.-J.; Li, H.-X.; Young, D. J.; Li, H.-Y.; Lang, J.-P. Reaction condition controlled nickel(II)-catalyzed C-C cross coupling of alcohols, *Org. Biomol. Chem.* **2019**, *17*, 3567–3574.

Figure S15. ^{1}H and ^{13}C NMR spectra of **3aa**

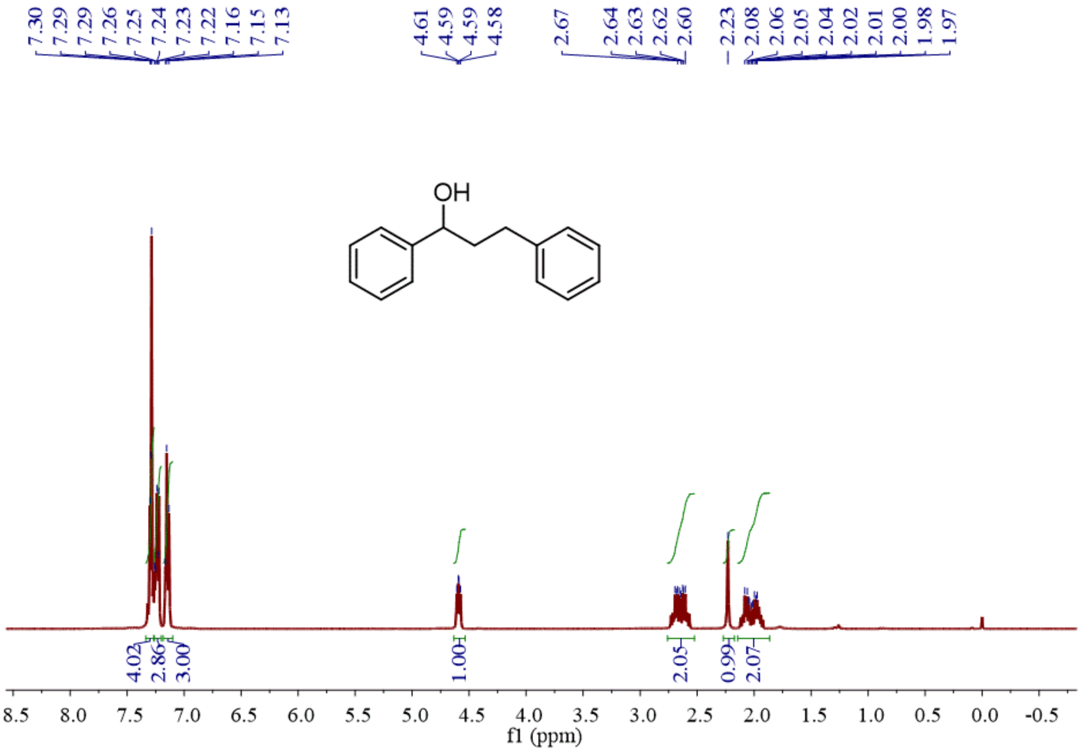

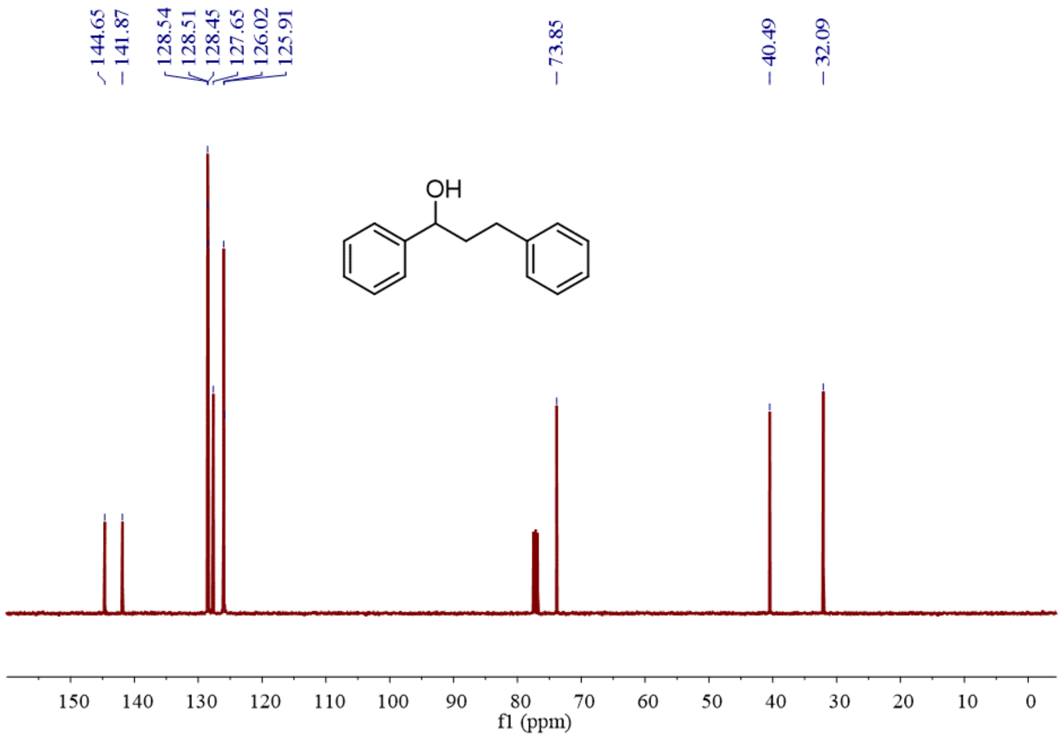

Figure S16. ^{1}H and ^{13}C NMR spectra of **3ab**

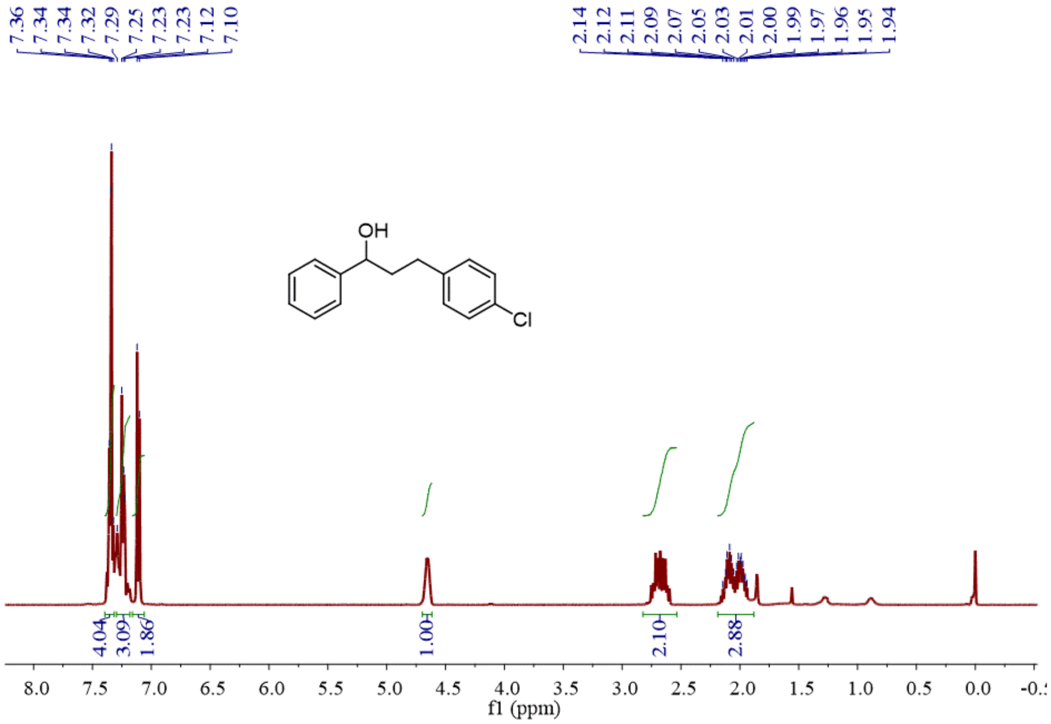

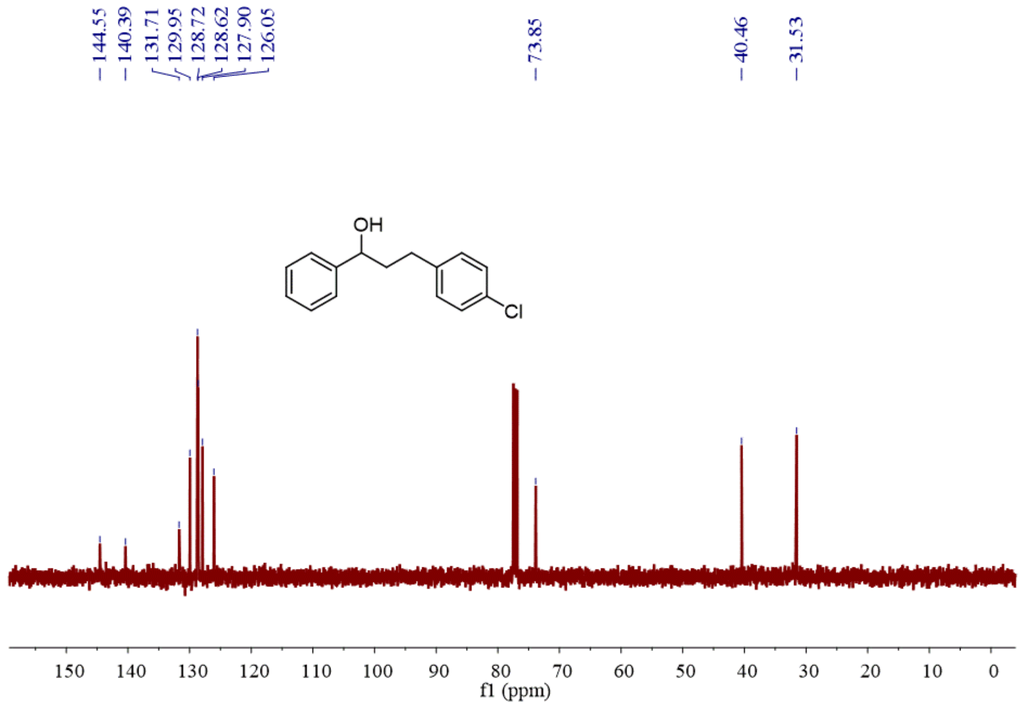

Figure S17. ^{1}H, ^{13}C and ^{19}F NMR spectra of **3ac**

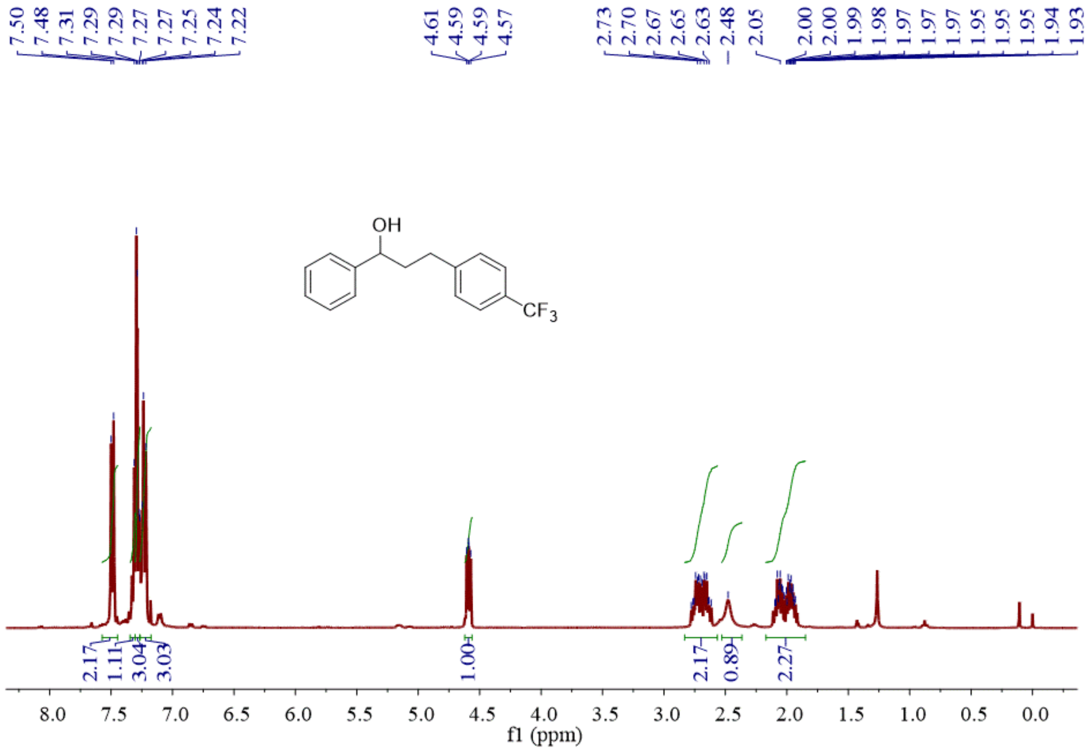

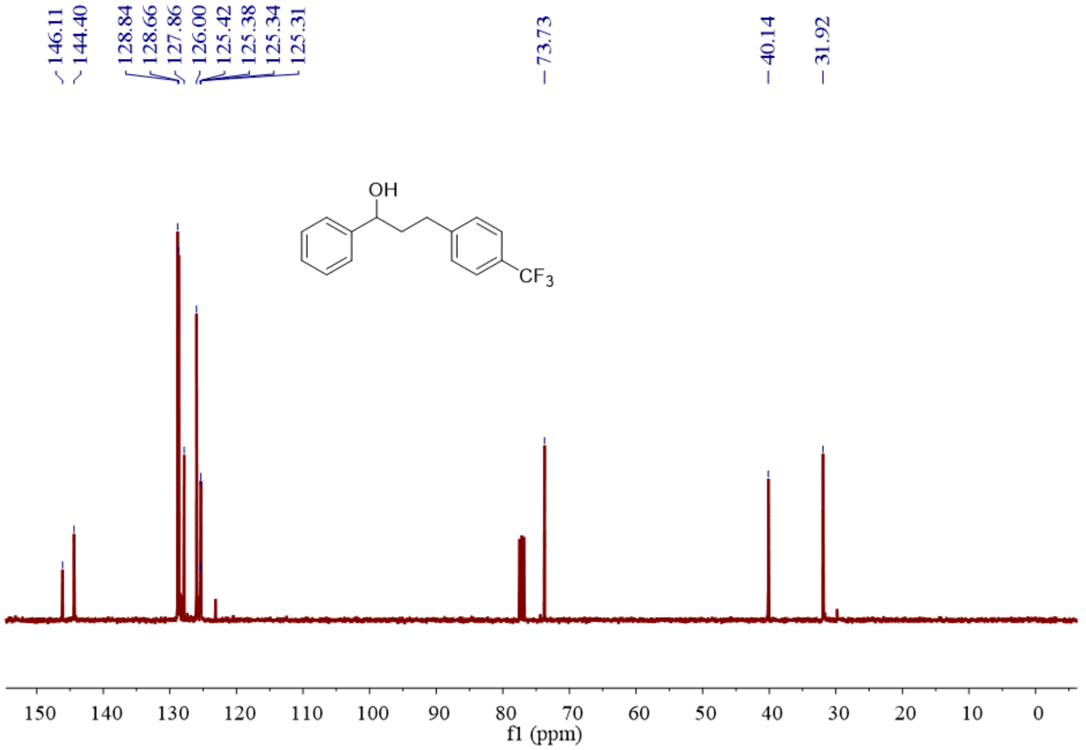

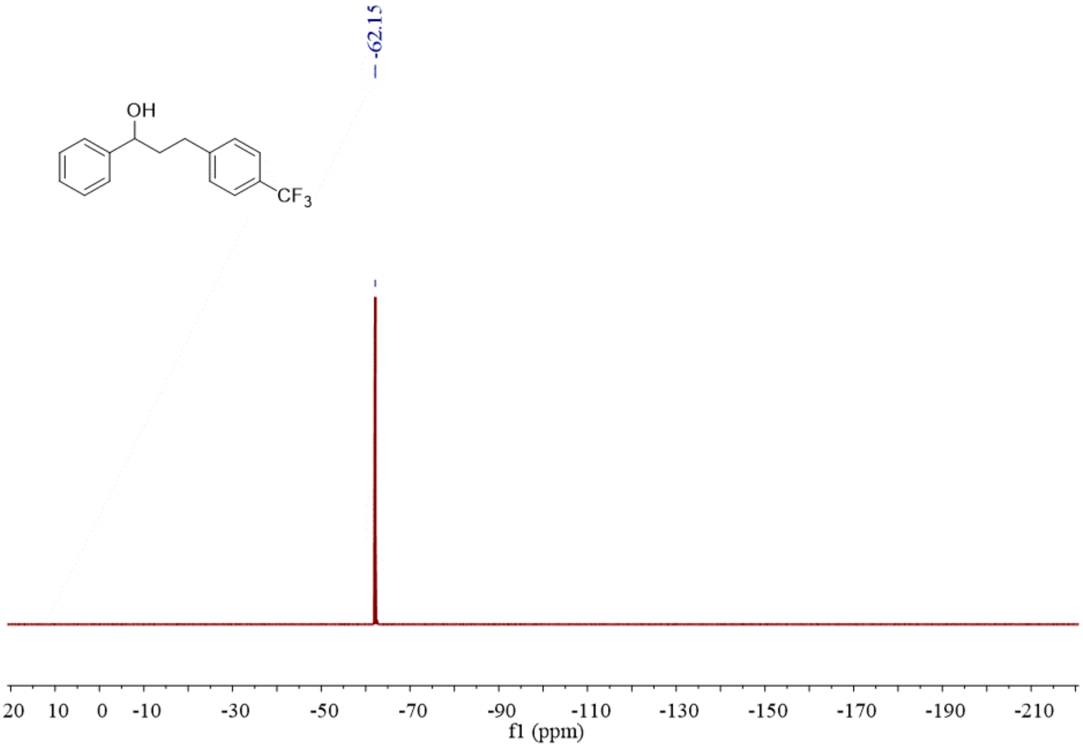
-62.15
OH
CF3
20
10
0
-10
-30
-50
-70
-90
-110
-130
-150
-170
-190
-210
f1 (ppm)

Figure S18. ^{1}H and ^{13}C NMR spectra of **3ad**

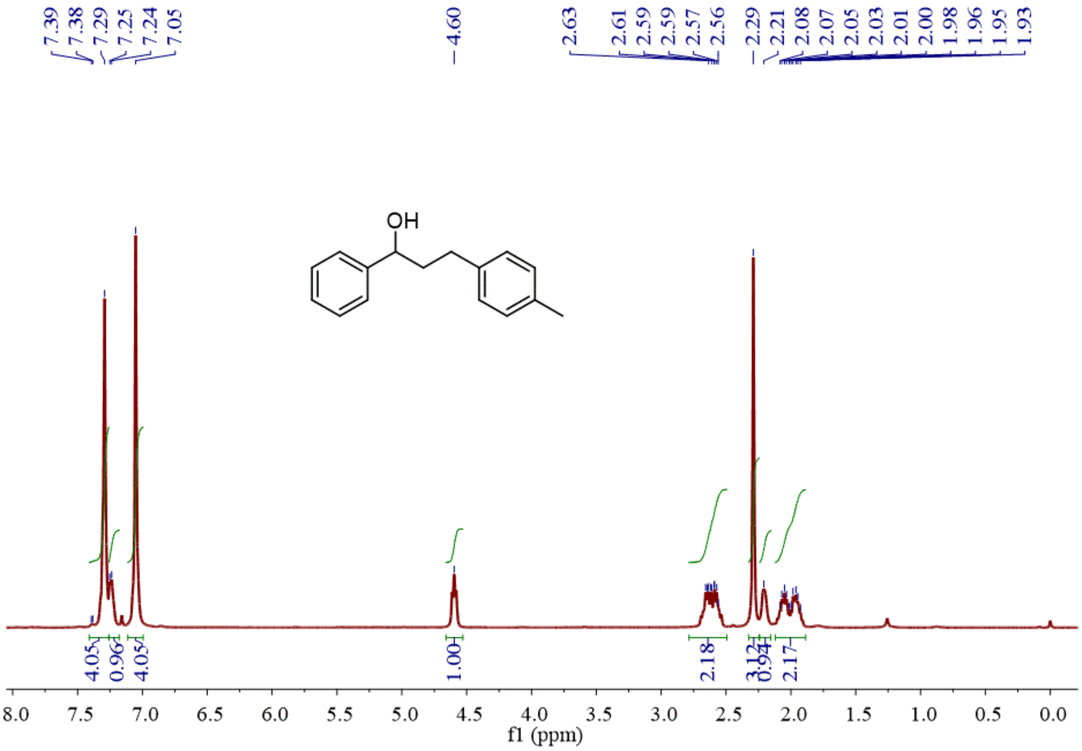

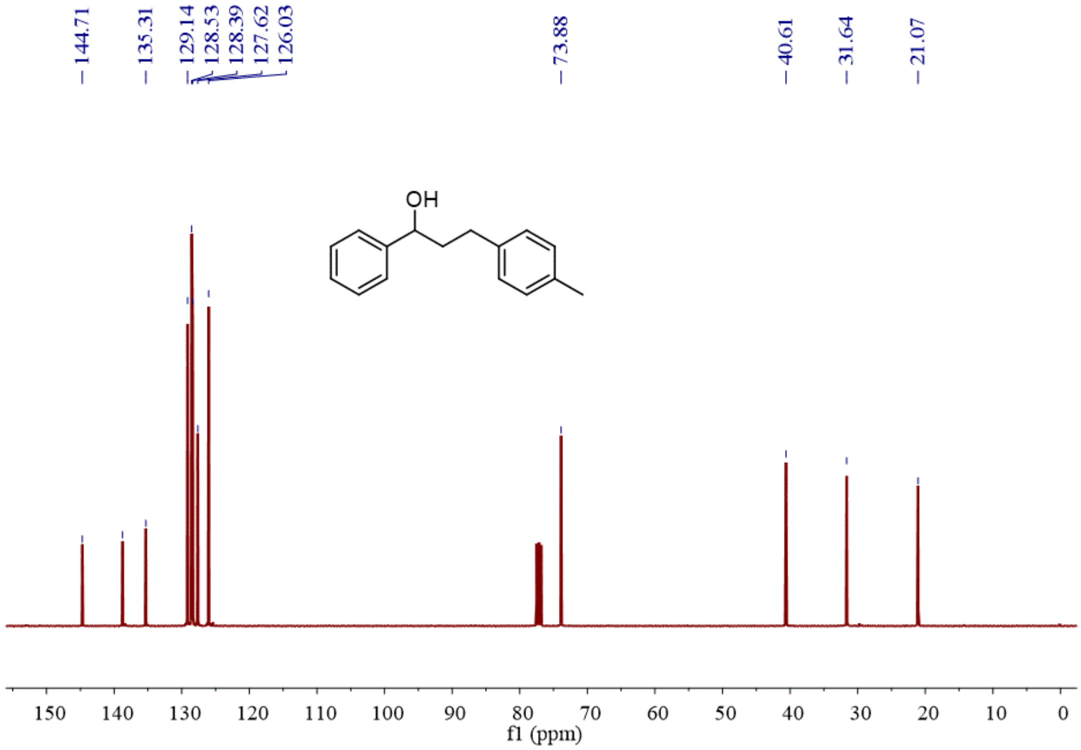

Figure S19. ^{1}H and ^{13}C NMR spectra of **3ae**

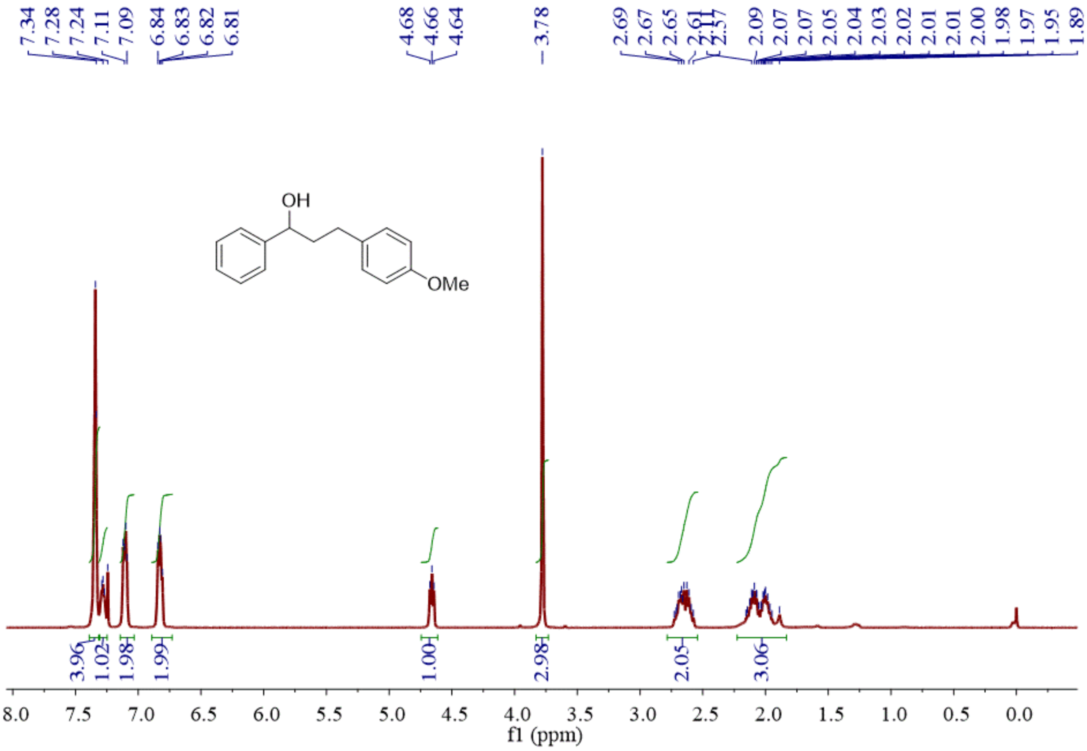

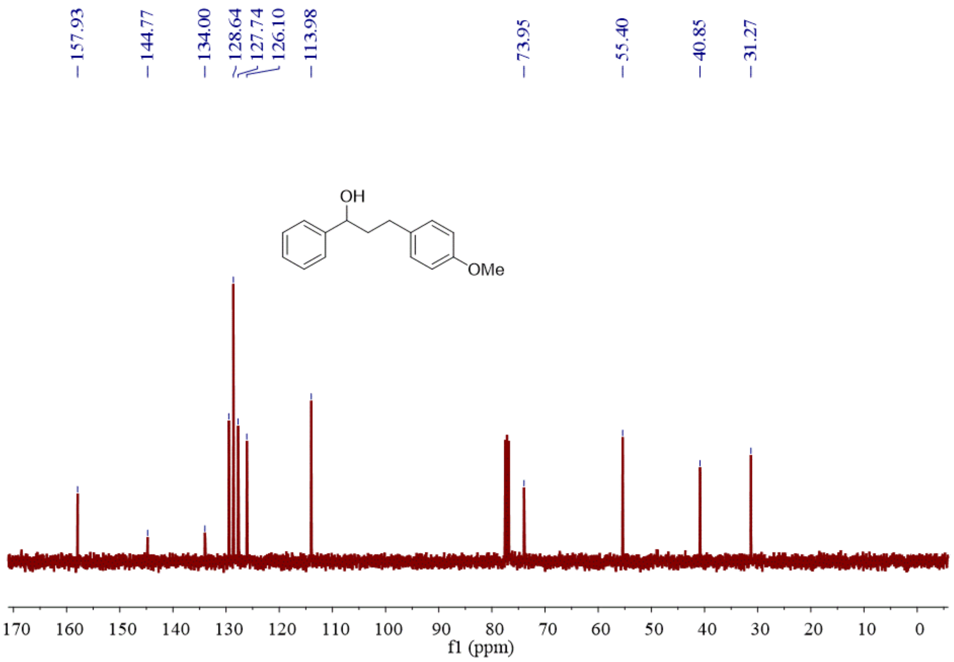

Figure S20. ^{1}H and ^{13}C NMR spectra of **3af**

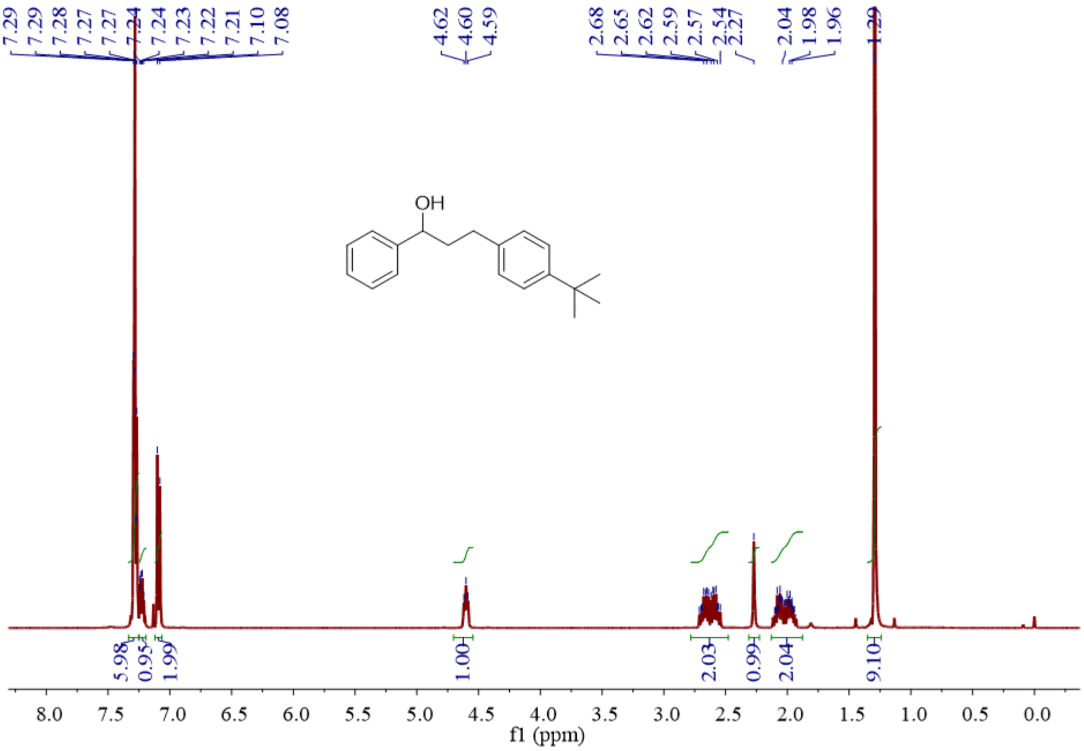

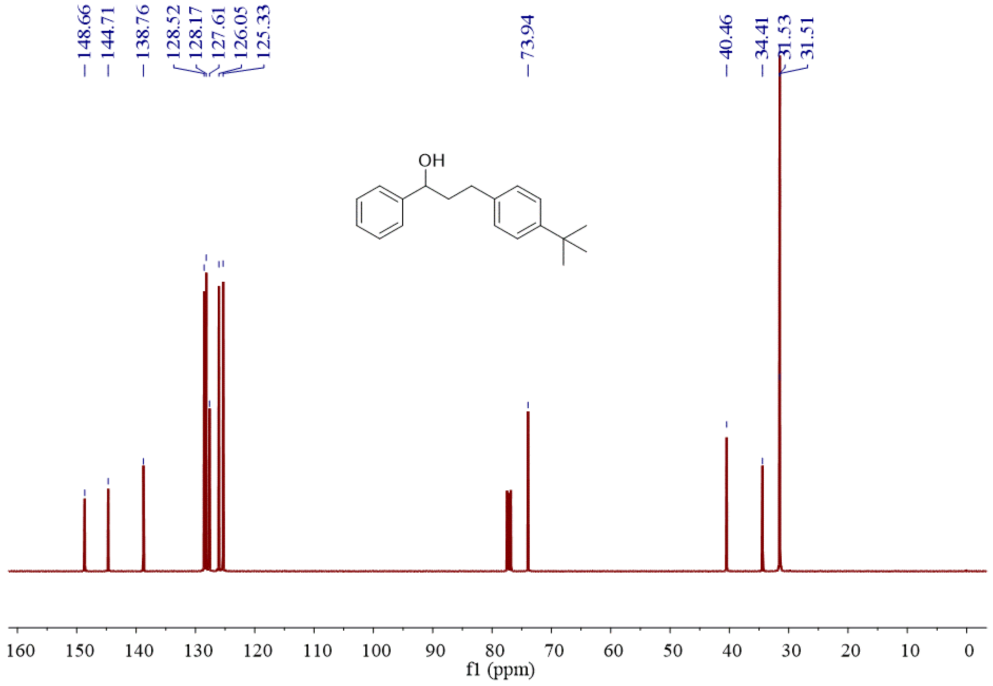

Figure S21. ^{1}H and ^{13}C NMR spectra of **3ag**

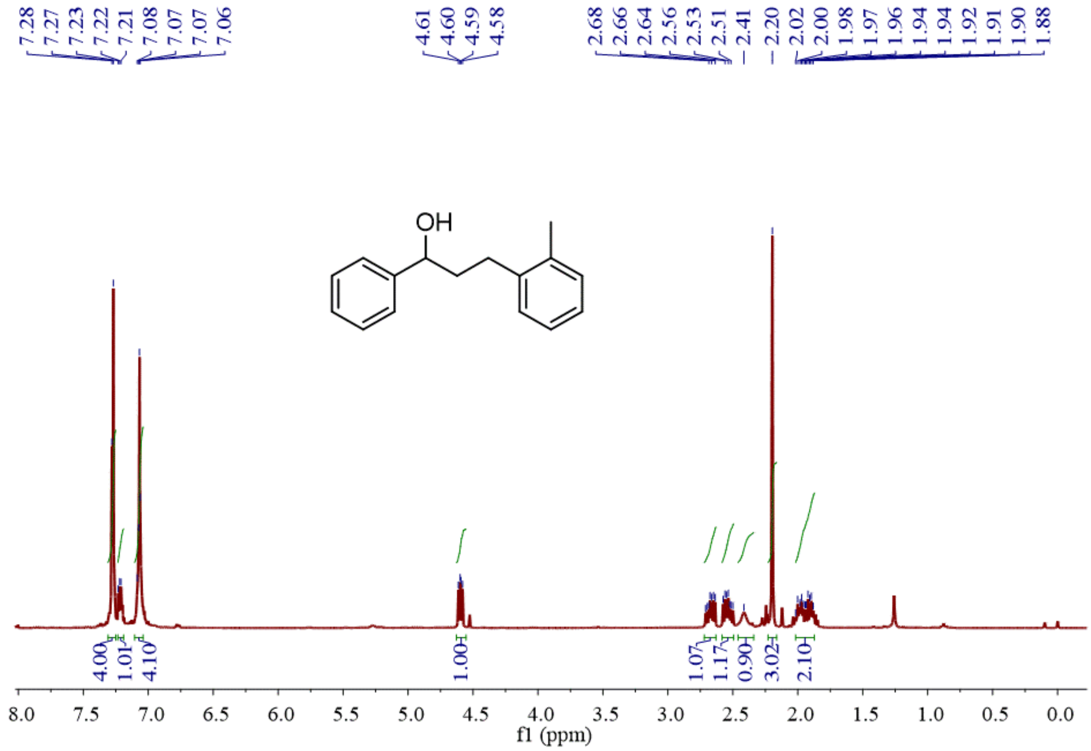

Figure S22. ^{1}H and ^{13}C NMR spectra of **3ah**

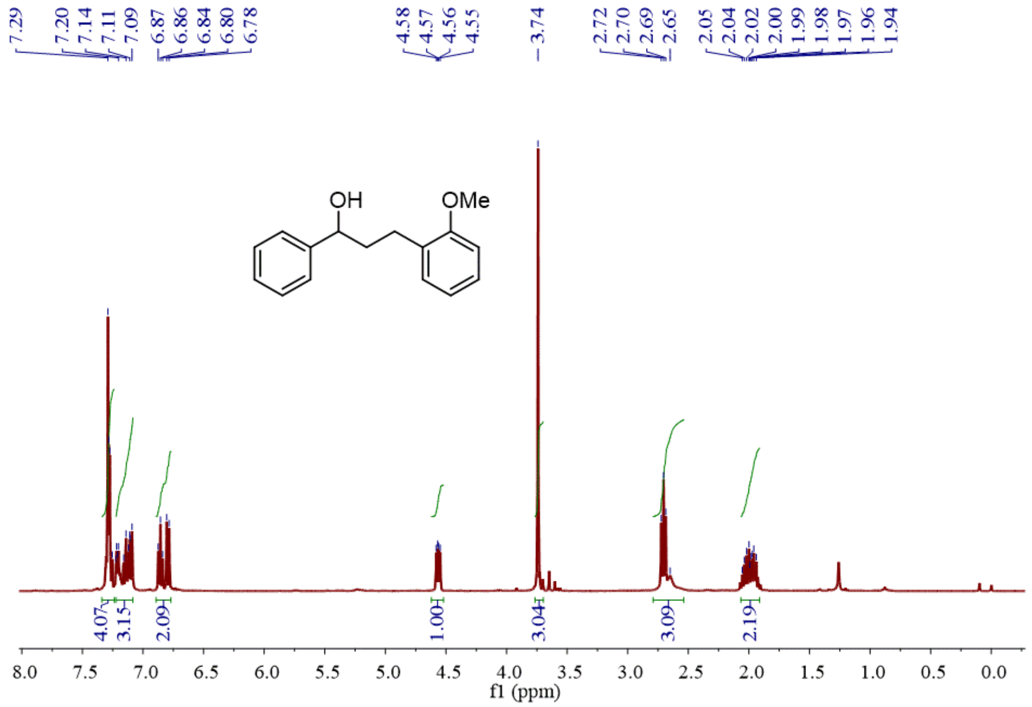

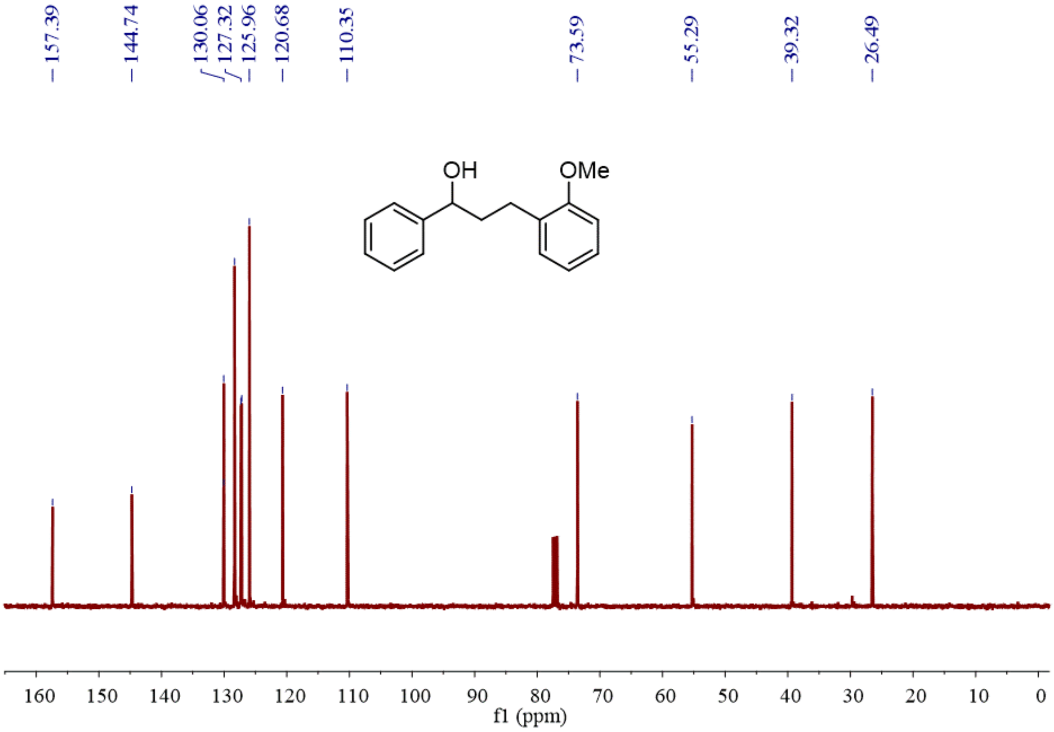

Figure S23. ^{1}H and ^{13}C NMR spectra of **3ai**

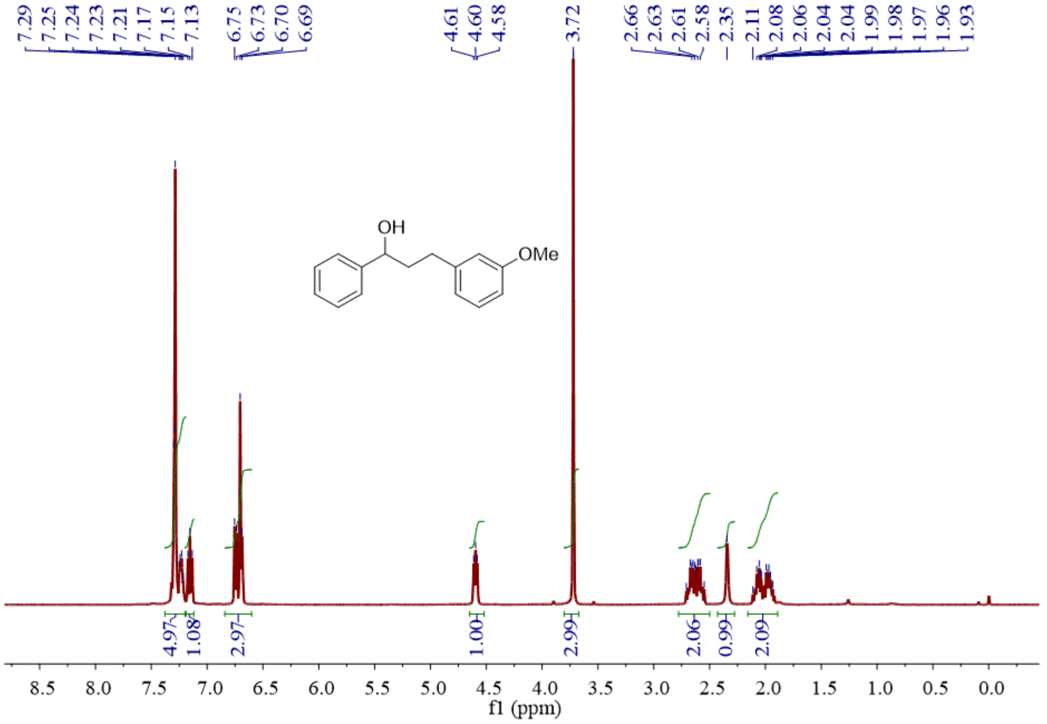

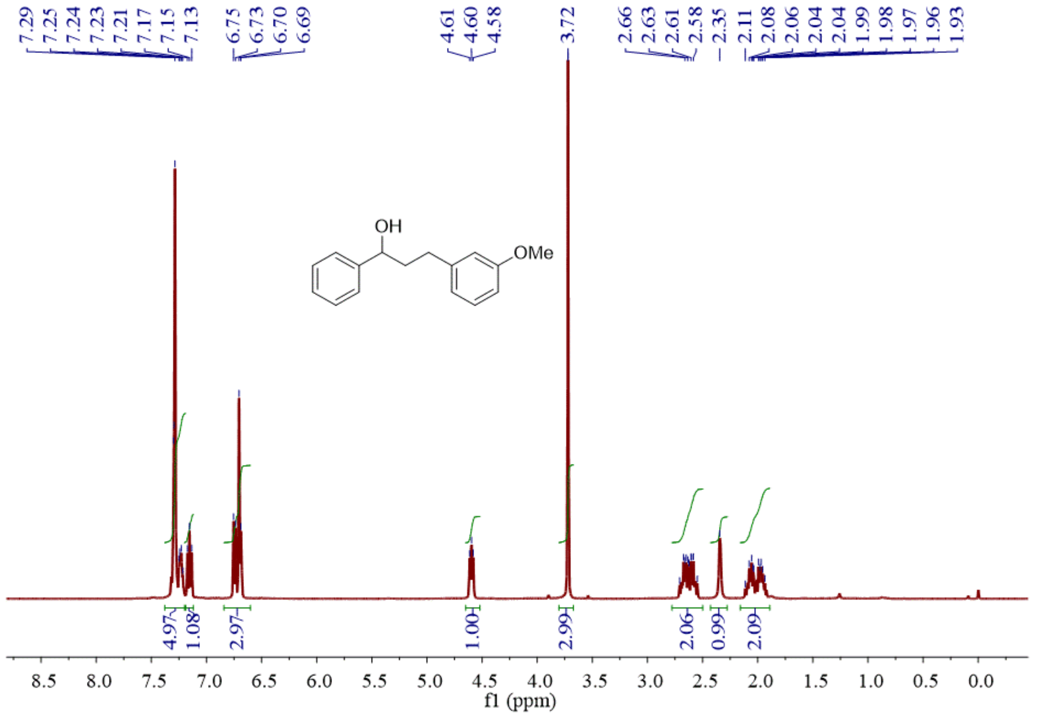

Figure S24. ^{1}H and ^{13}C NMR spectra of **3aj**

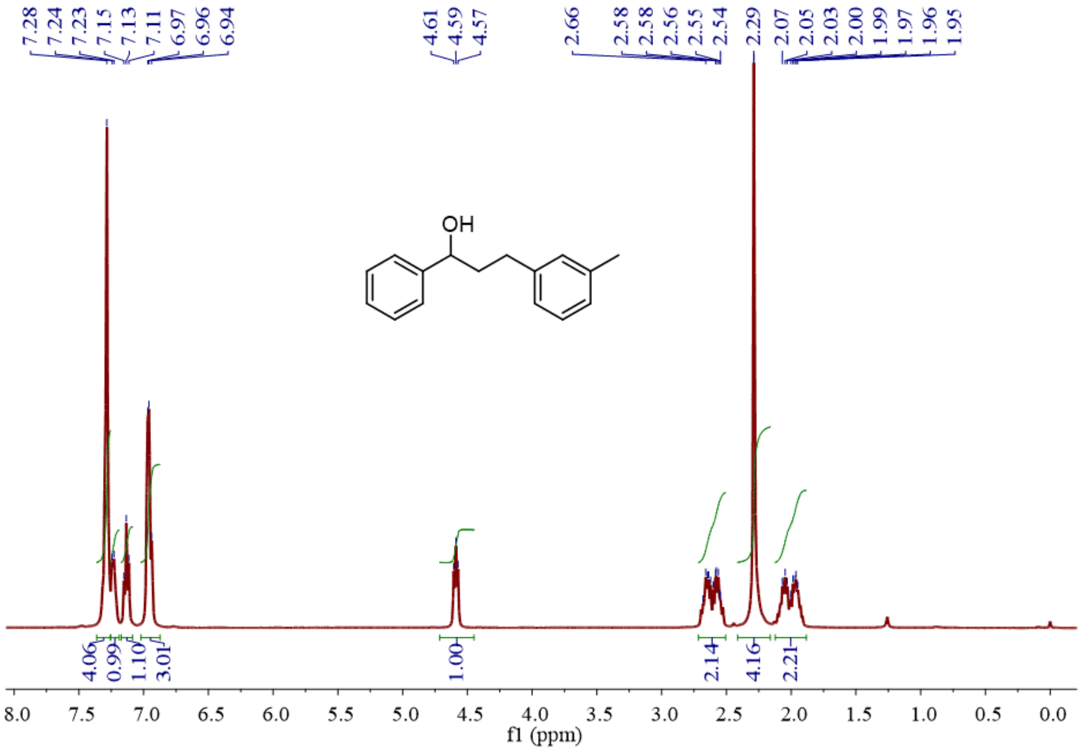

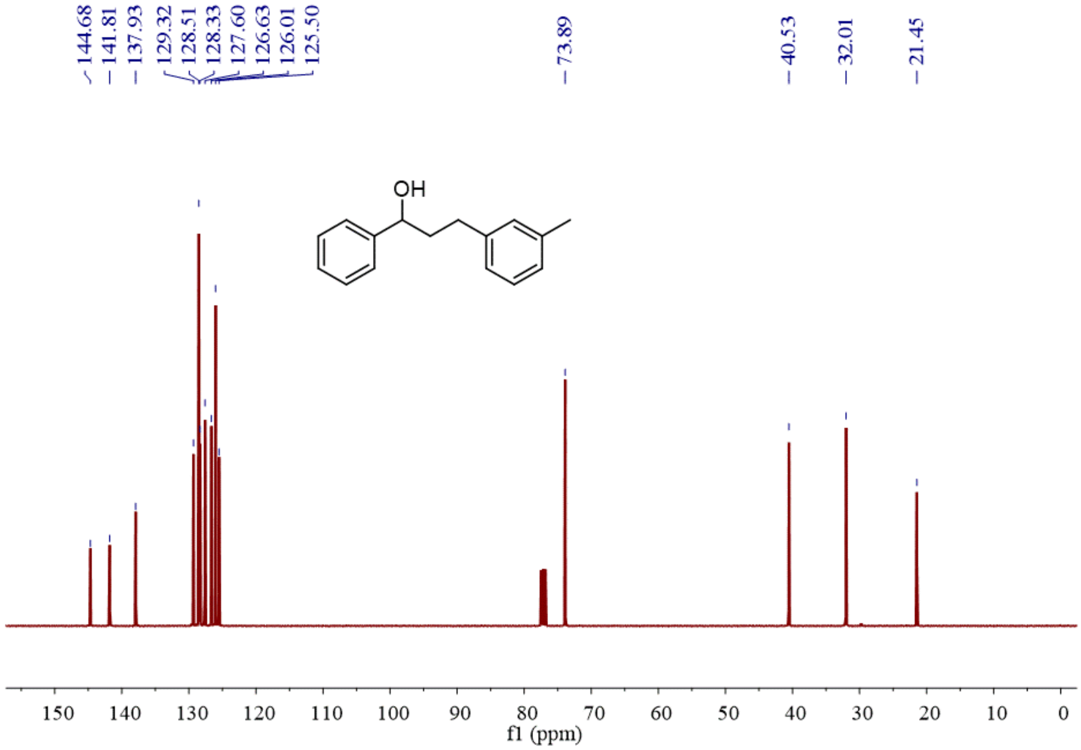

Figure S25. ^{1}H and ^{13}C NMR spectra of **3ak**

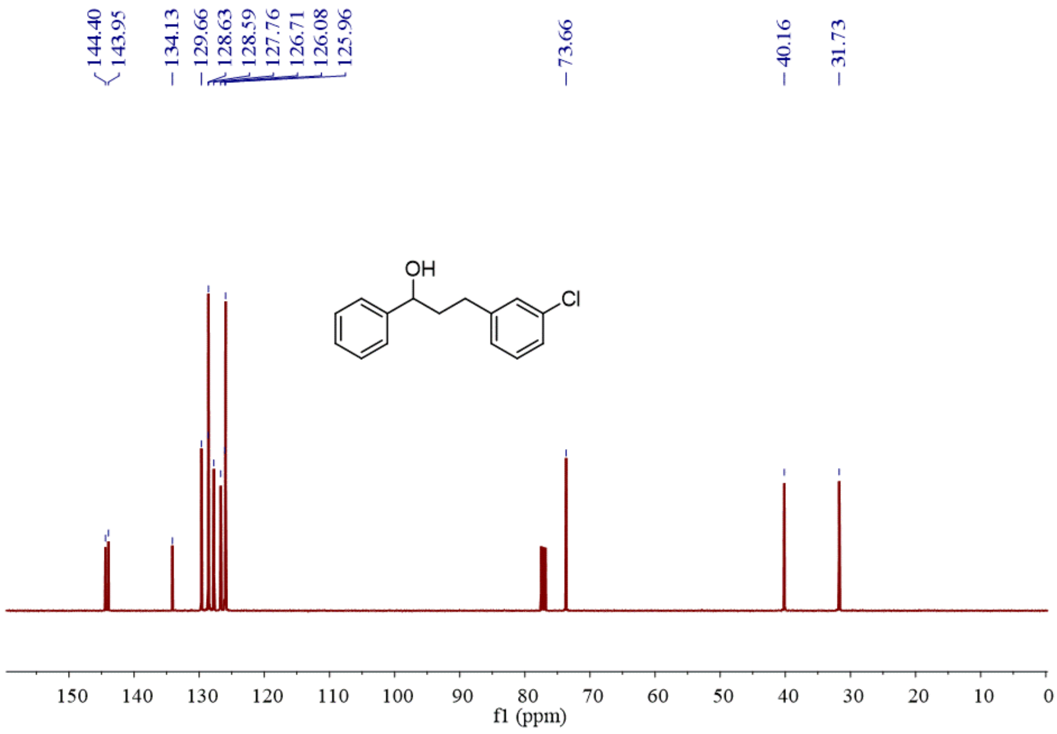

Figure S26. ^{1}H and ^{13}C NMR spectra of **3al**

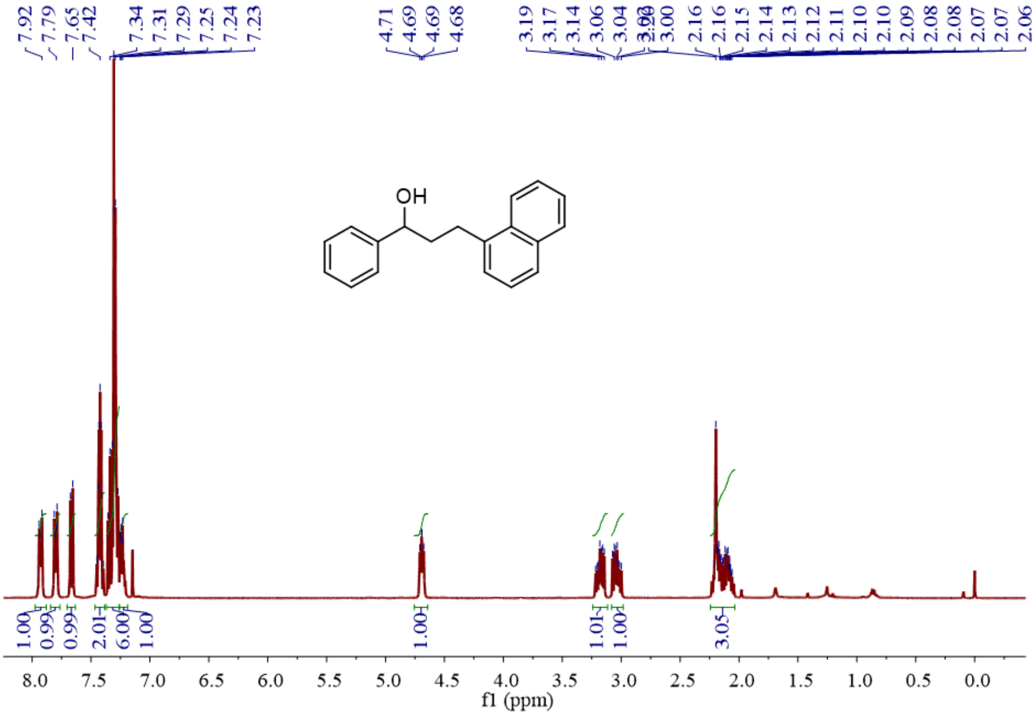

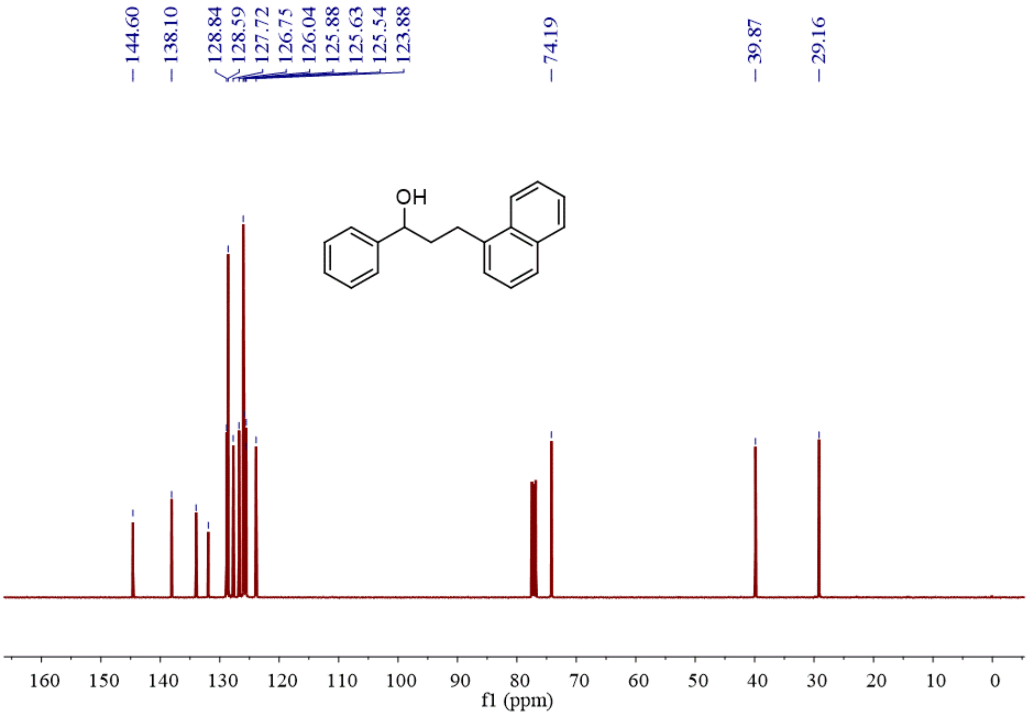

Figure S27. ^{1}H and ^{13}C NMR spectra of **3am**

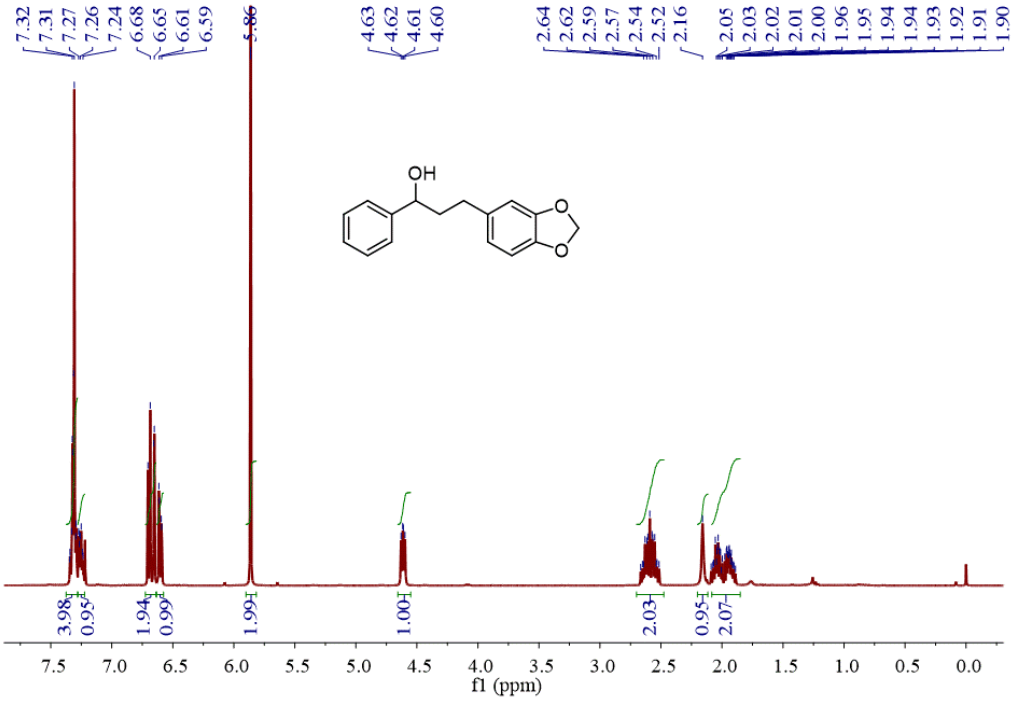

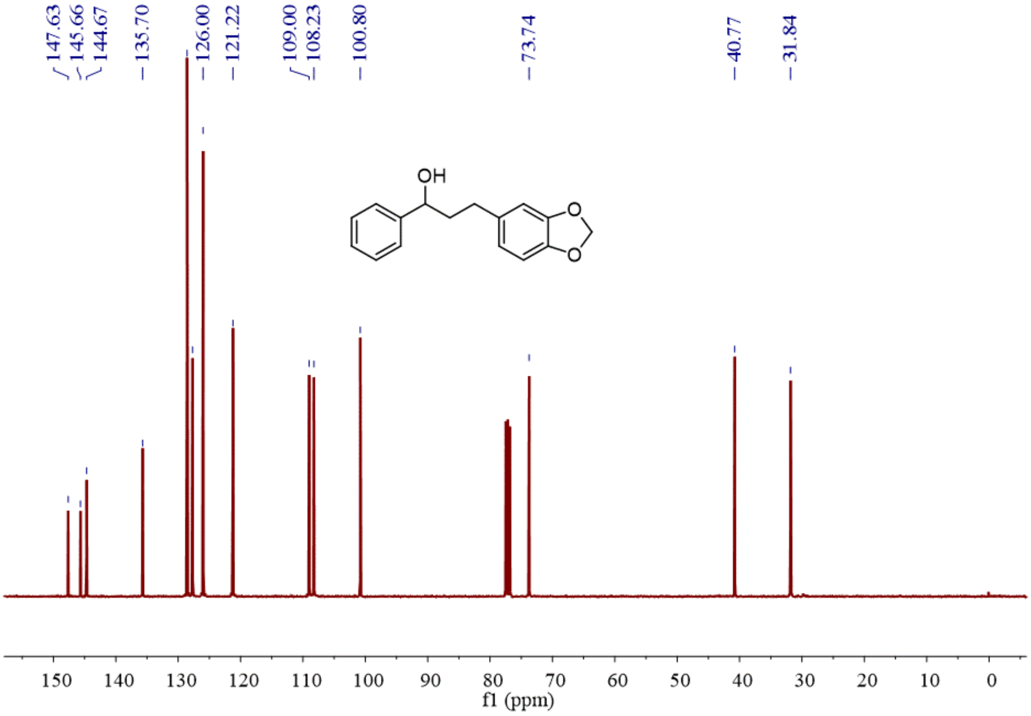

Figure S28. ^{1}H and ^{13}C NMR spectra of **3an**

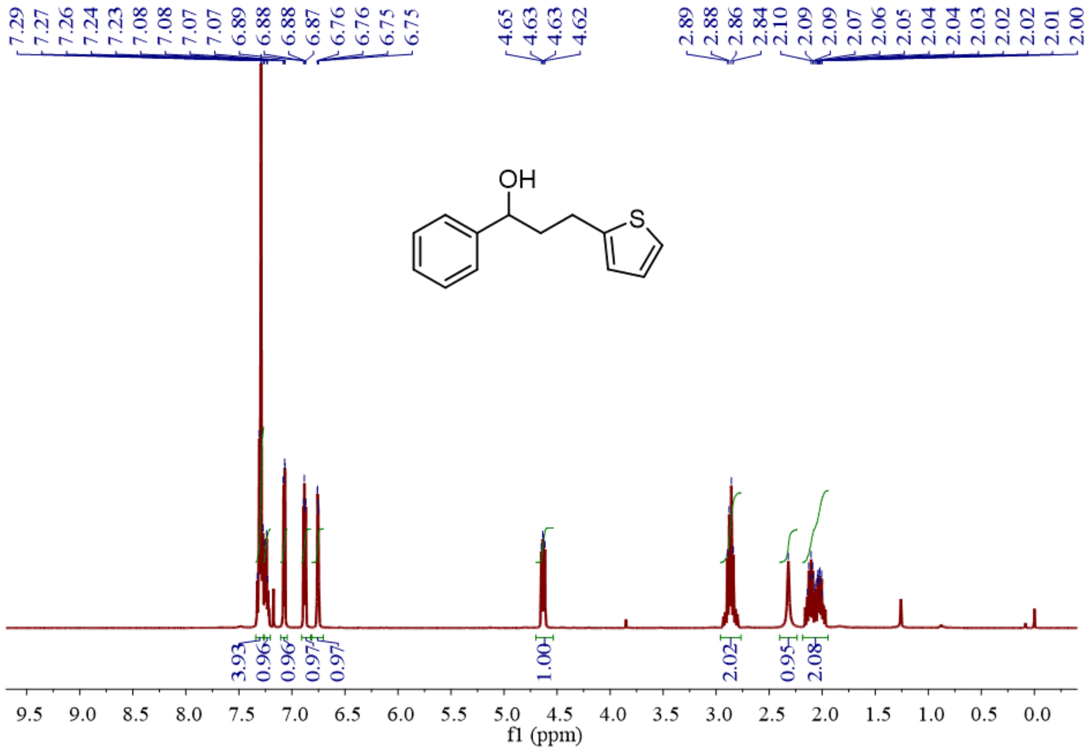

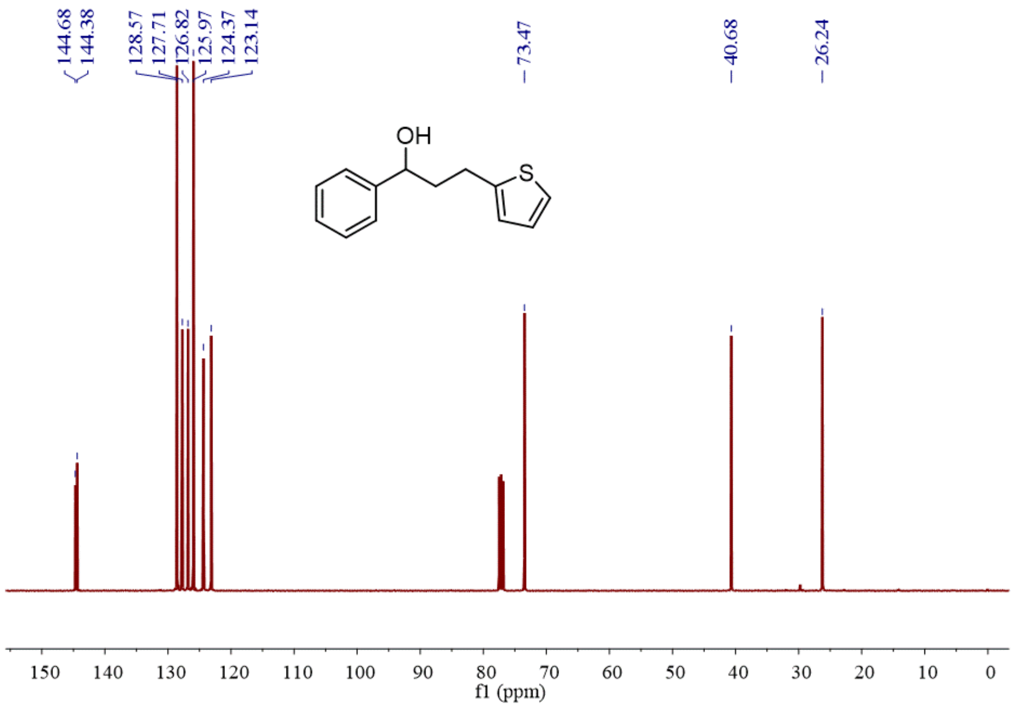

Figure S29. ^{1}H and ^{13}C NMR spectra of **3ba**

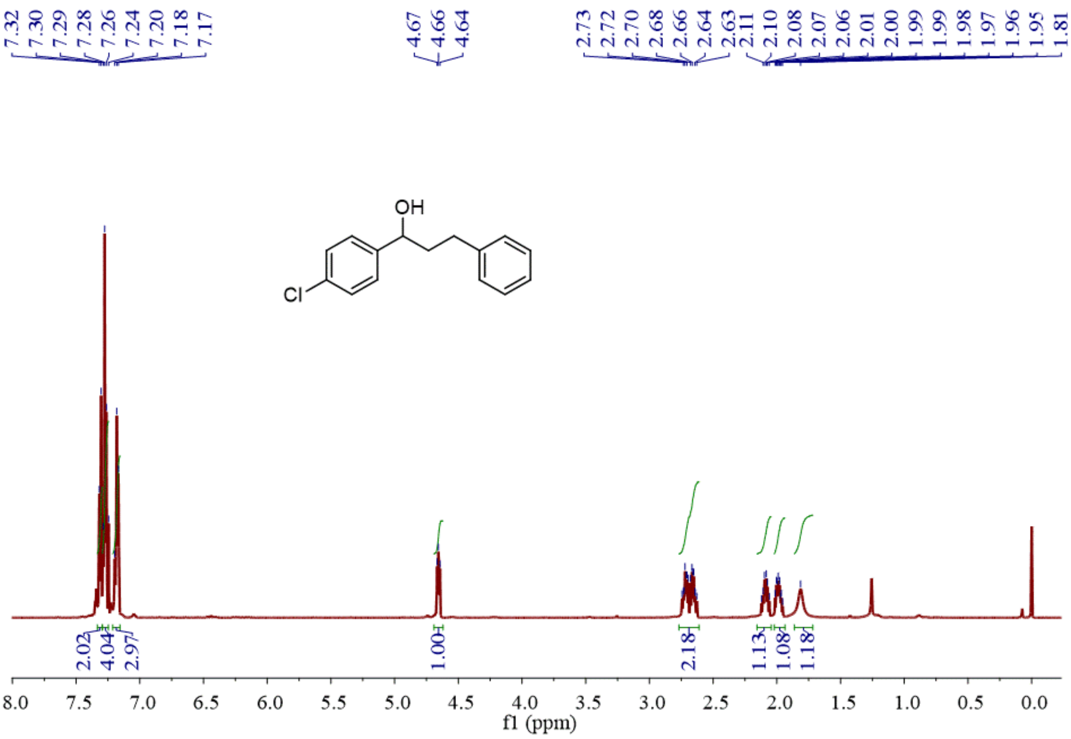

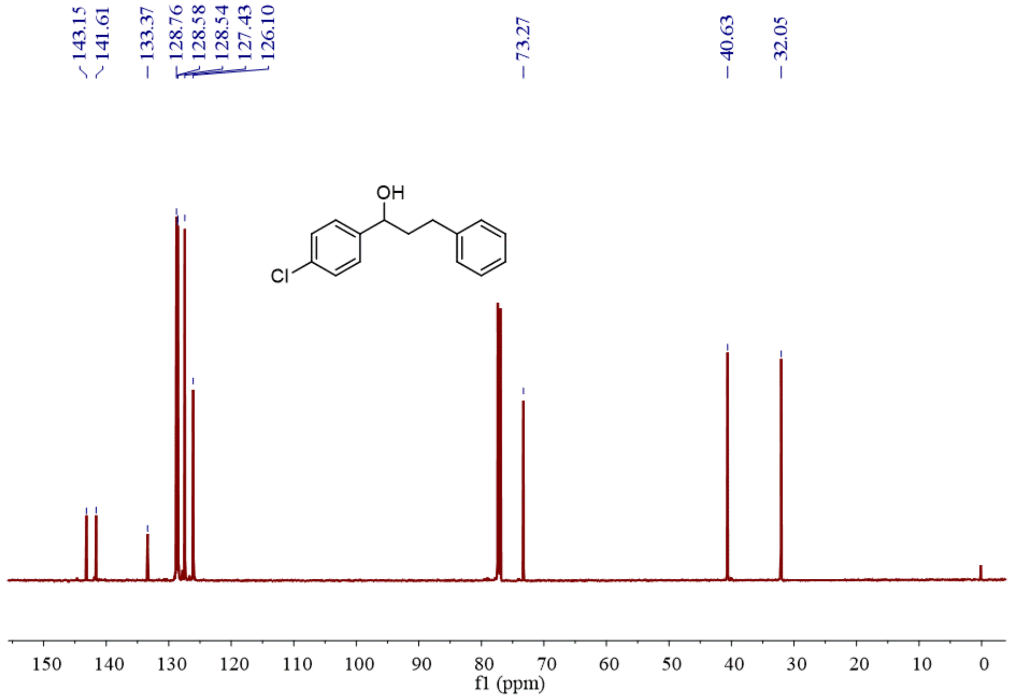

Figure S30. ^{1}H and ^{13}C NMR spectra of **3ca**

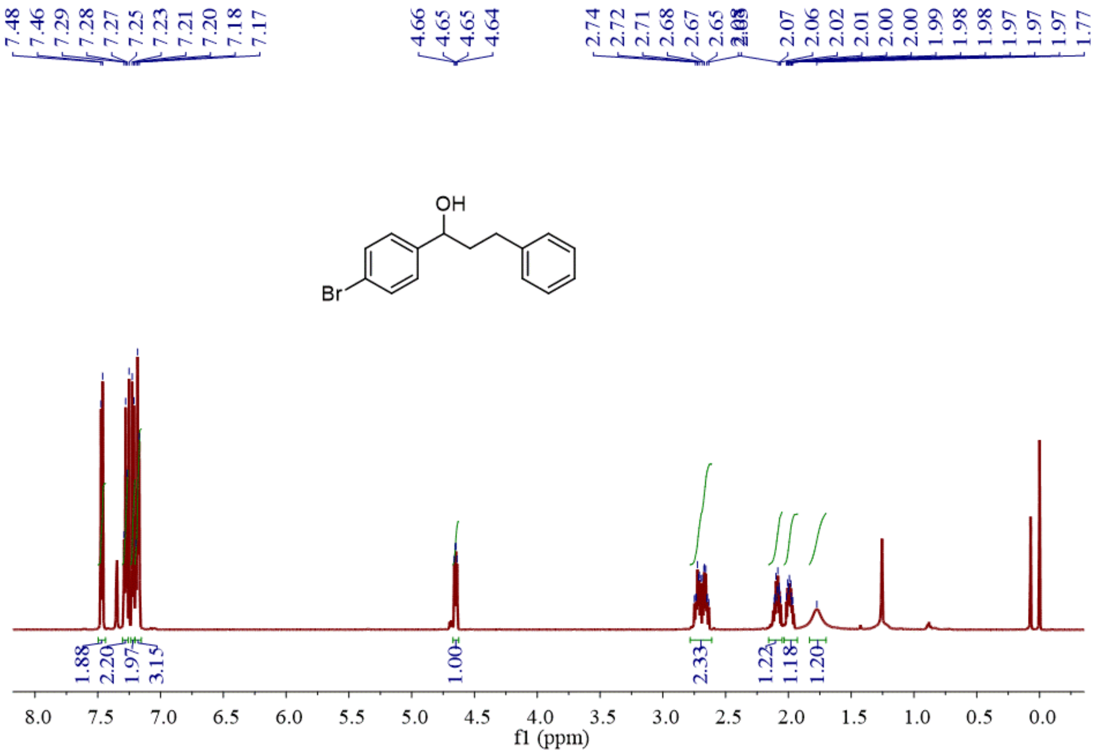

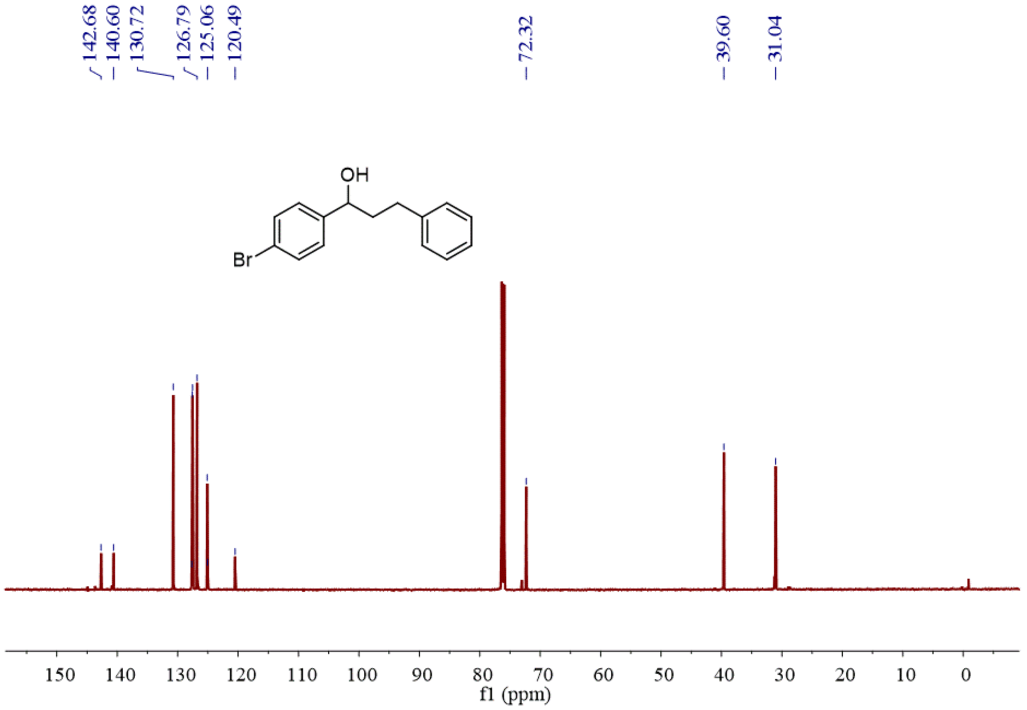

Figure S31. ^{1}H and ^{13}C NMR spectra of **3da**

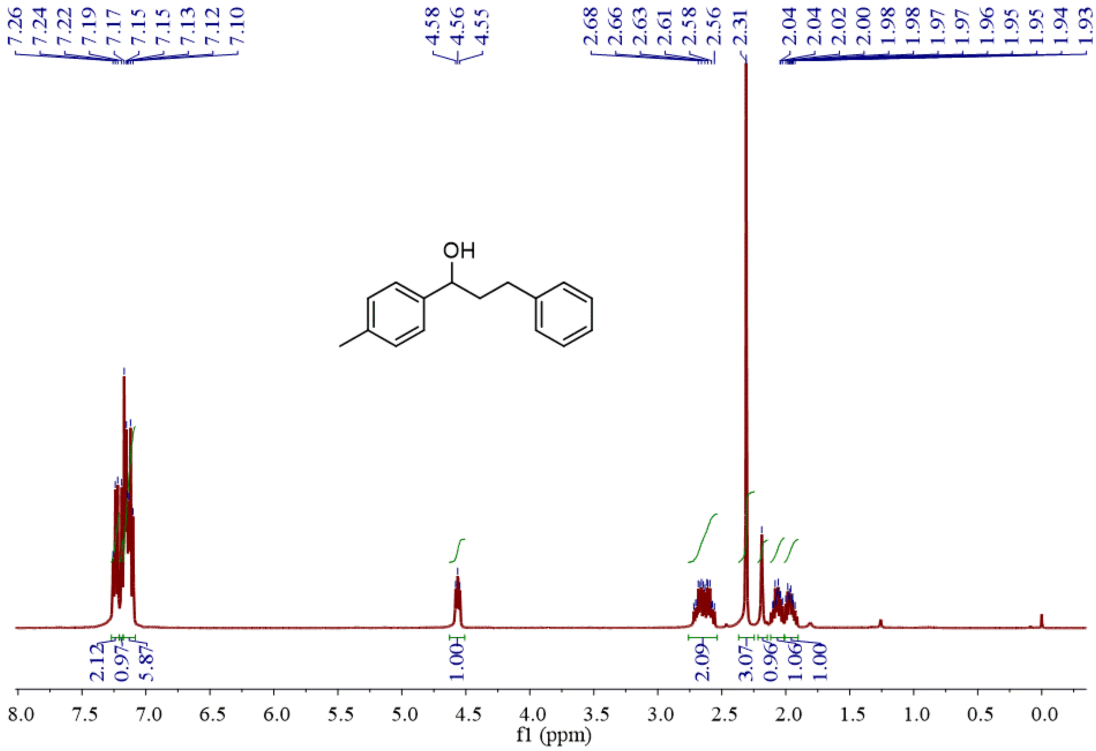

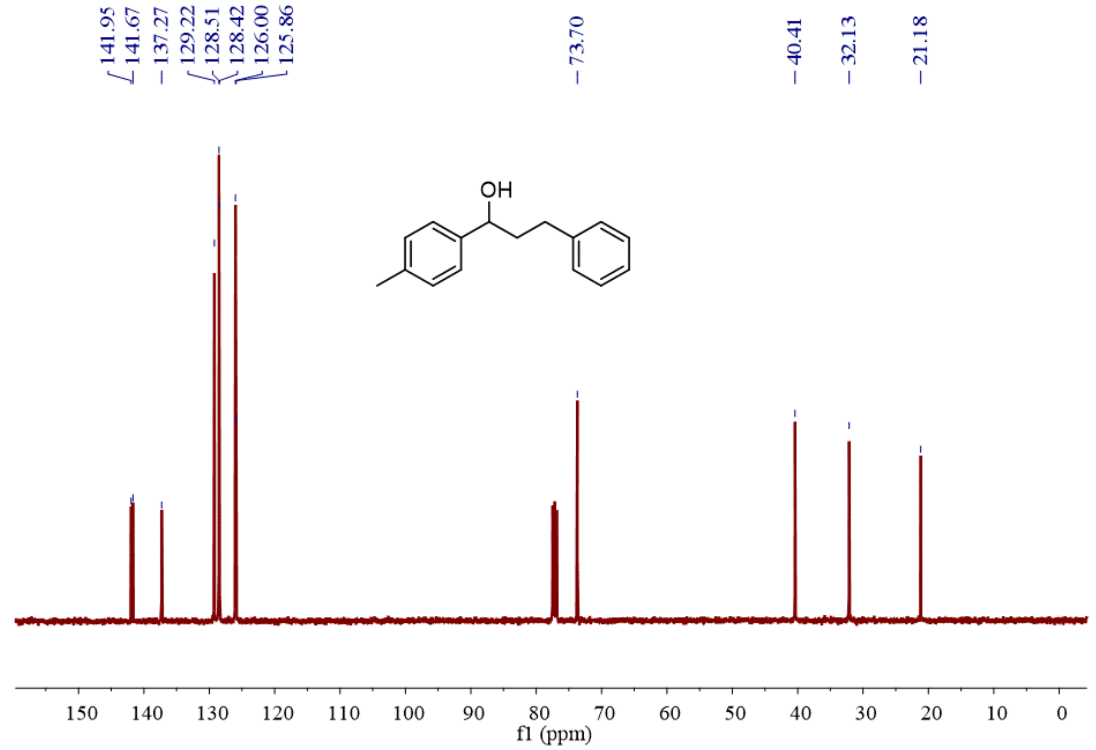

Figure S32. ^{1}H and ^{13}C NMR spectra of **3ea**

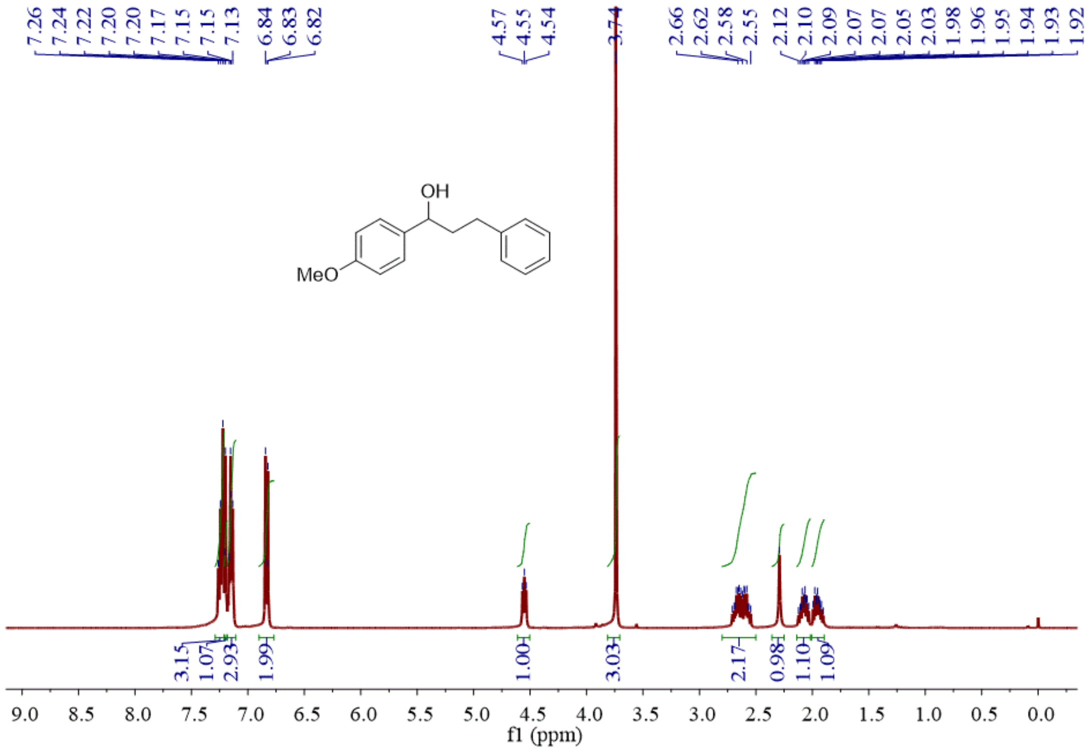

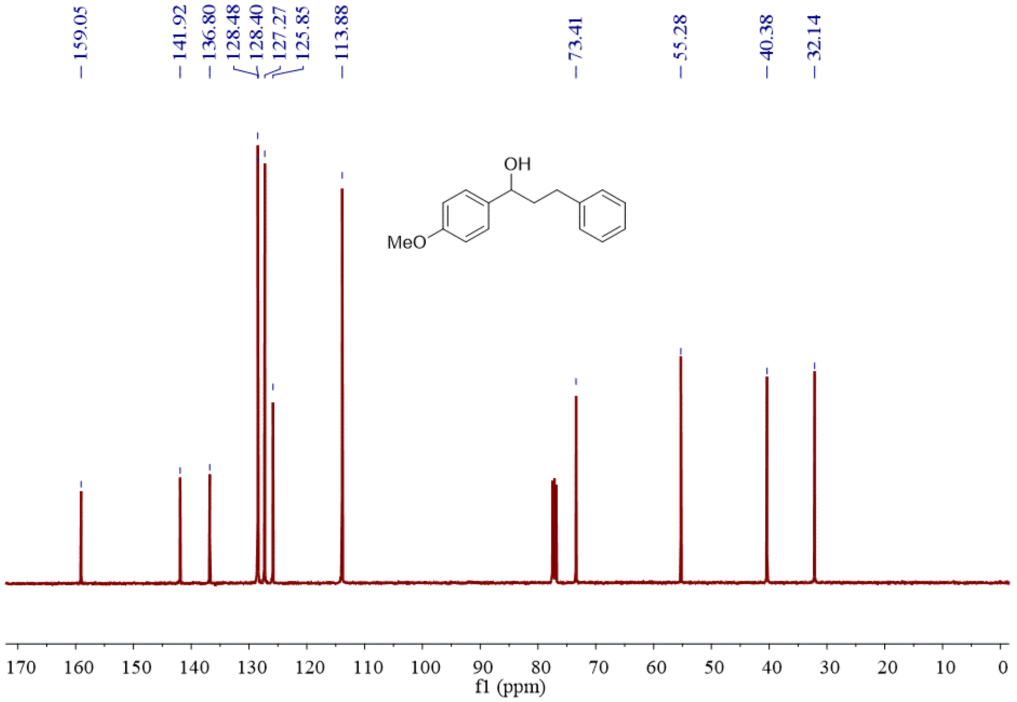

Figure S33. ^{1}H and ^{13}C NMR spectra of **3fa**

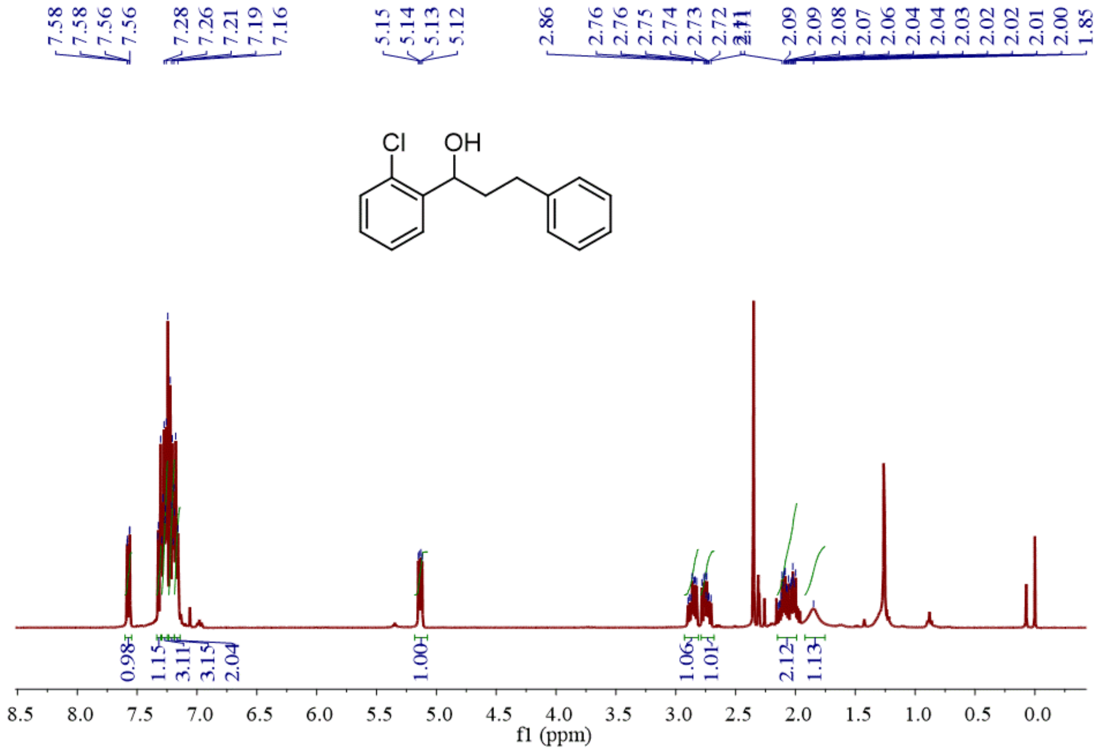

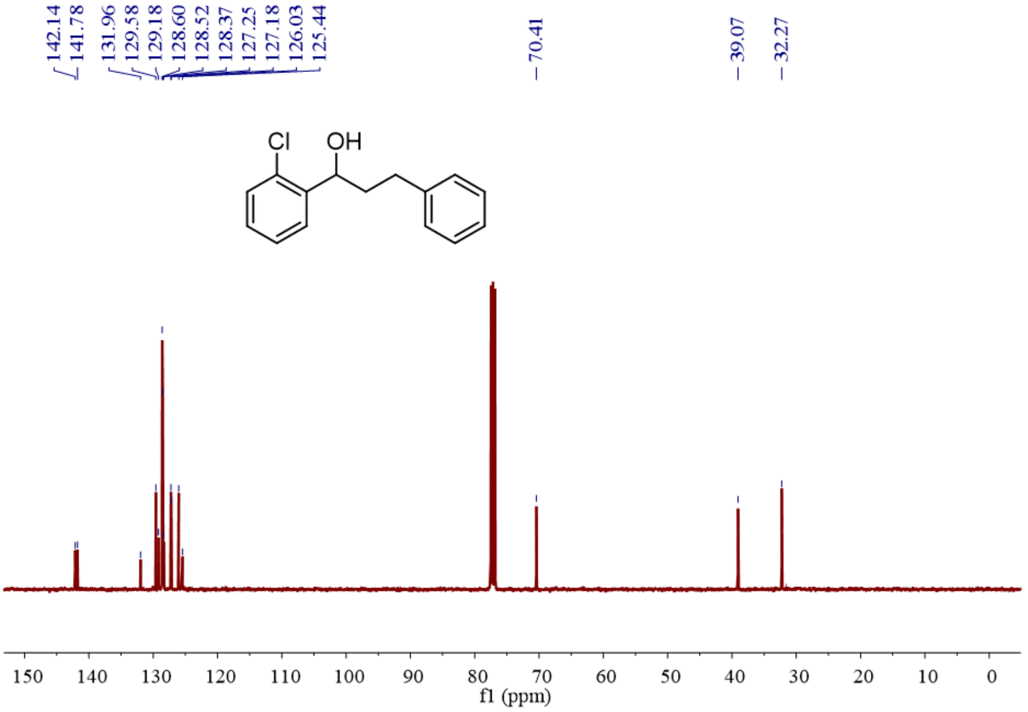

Figure S34 ^{1}H and ^{13}C NMR spectra of **3ga**

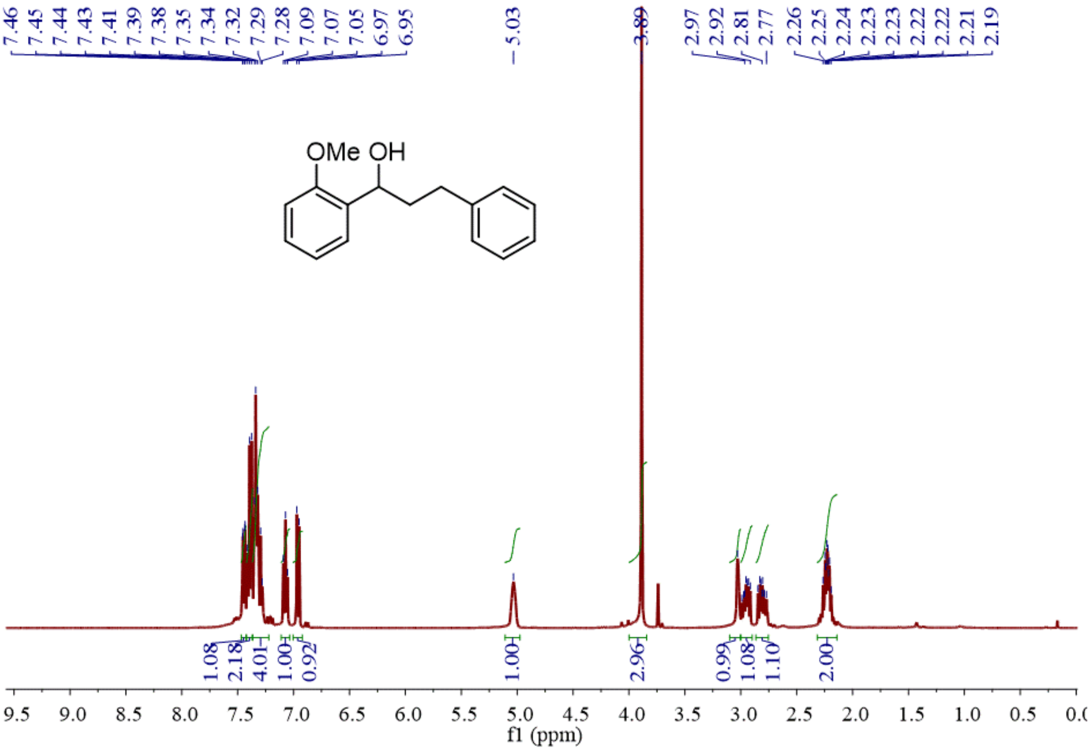

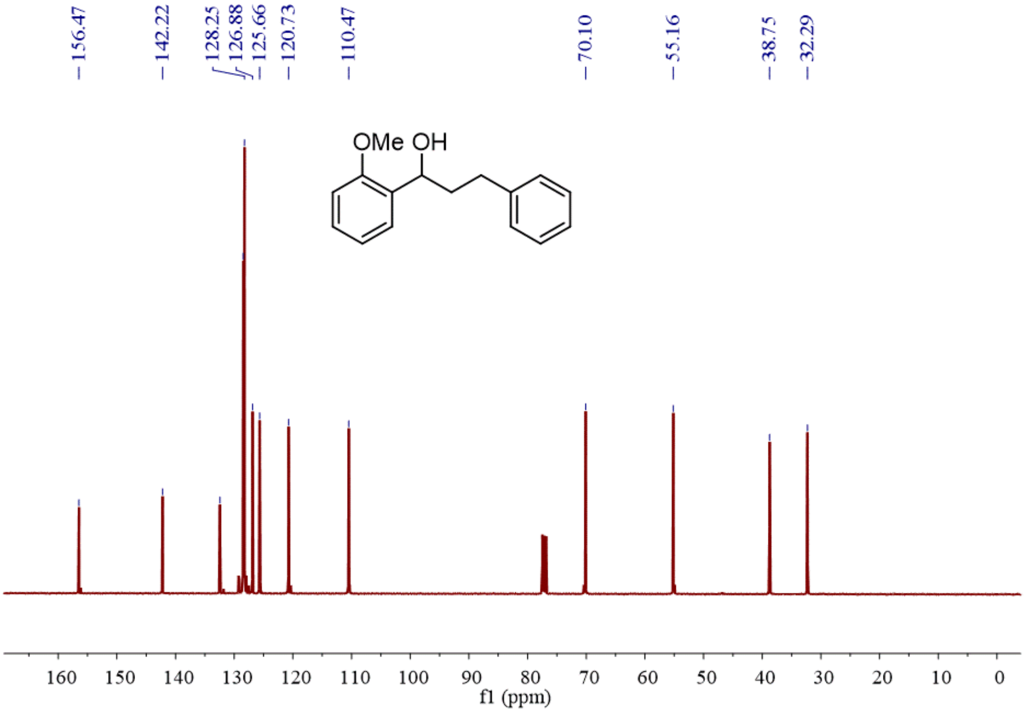

Figure S35. ^{1}H and ^{13}C NMR spectra of **3ha**

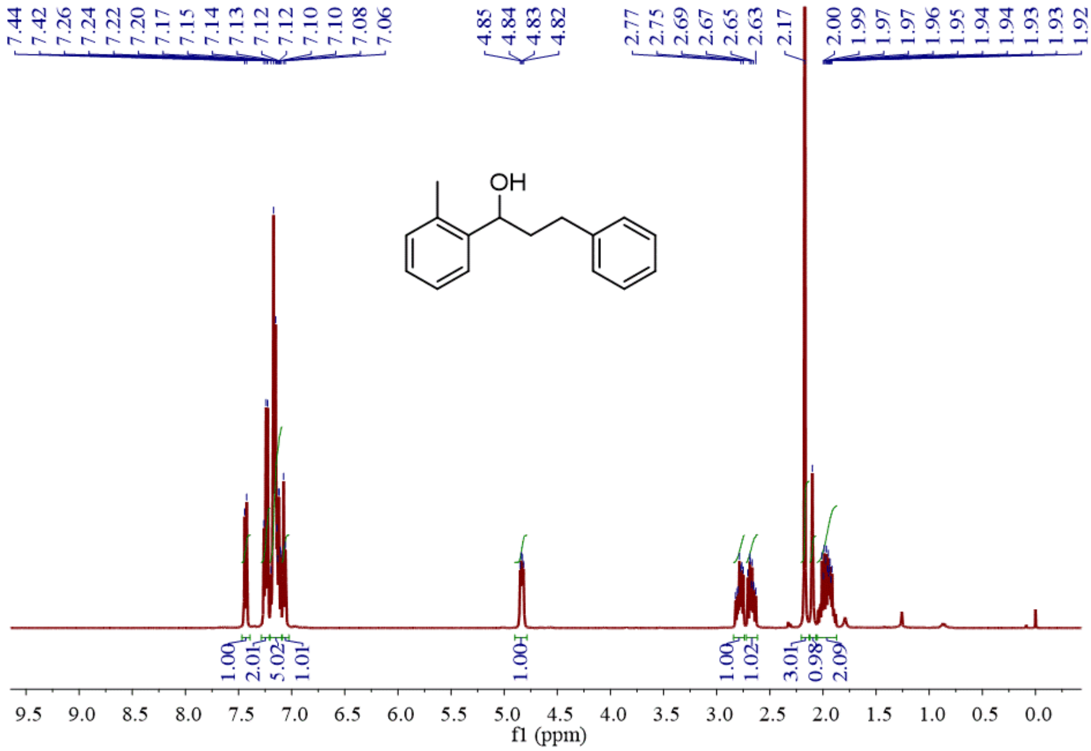

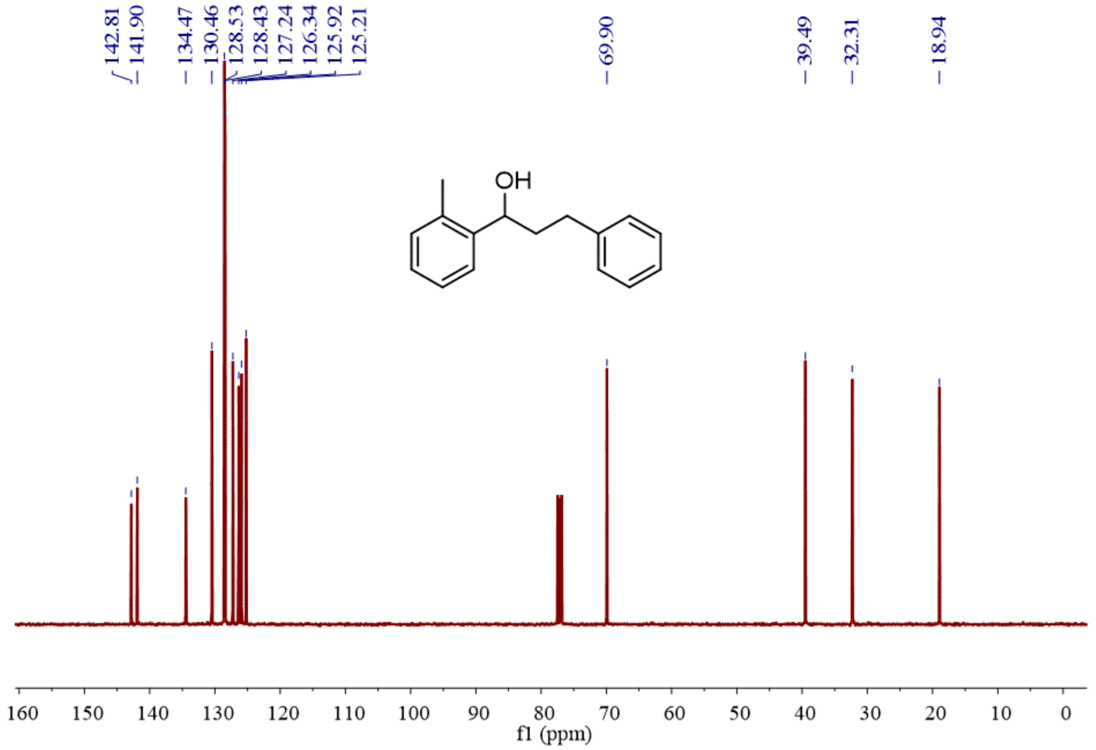

Figure S36. ^{1}H and ^{13}C NMR spectra of **3ia**

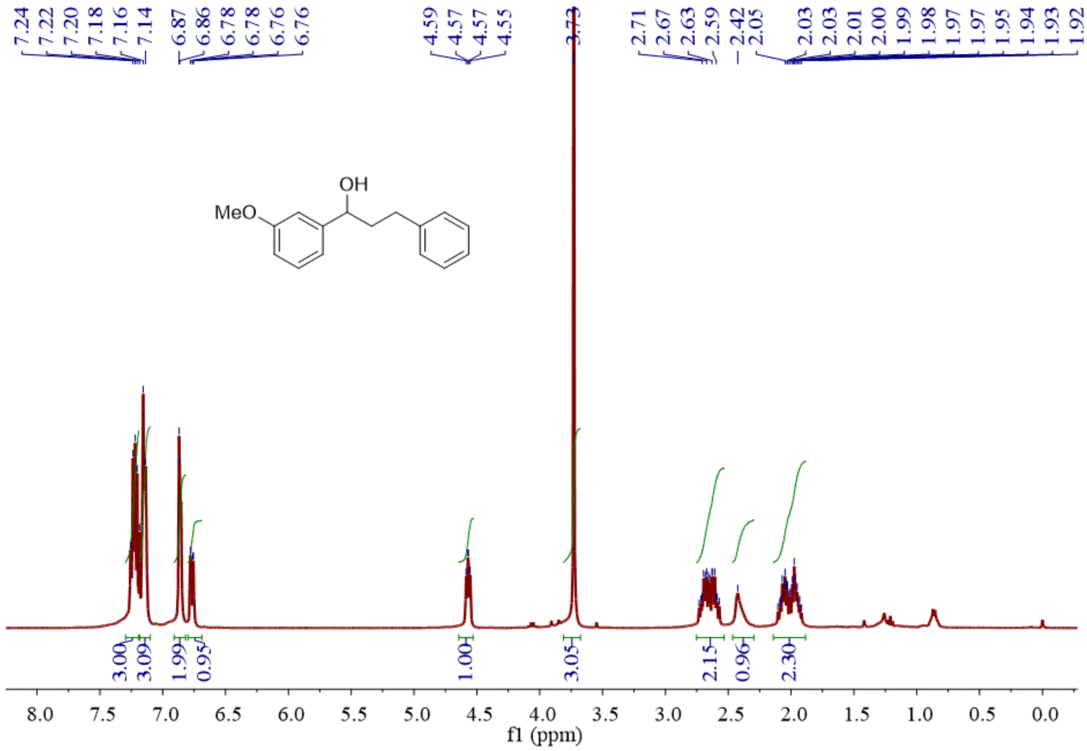

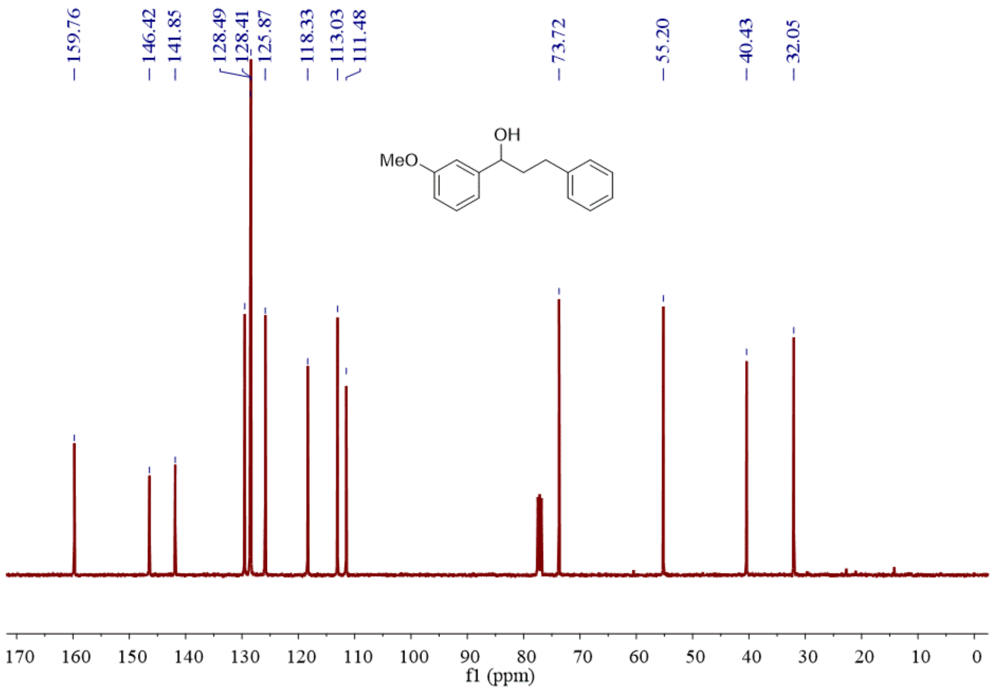

Figure S37. ^{1}H and ^{13}C NMR spectra of **3ja**

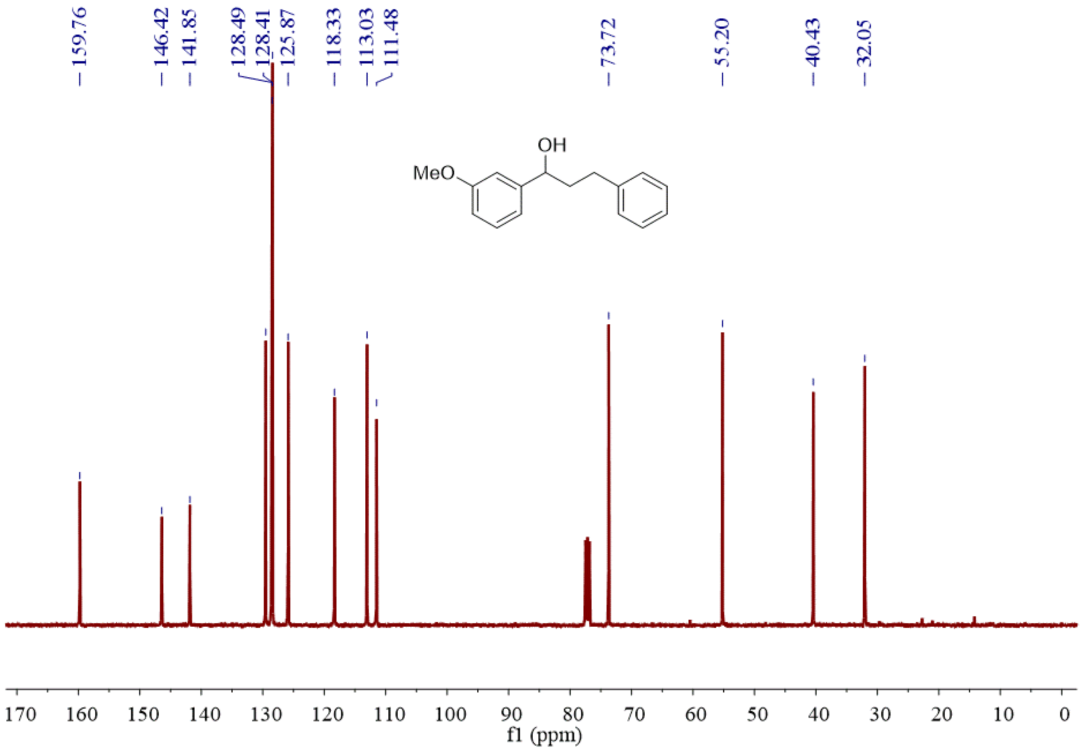

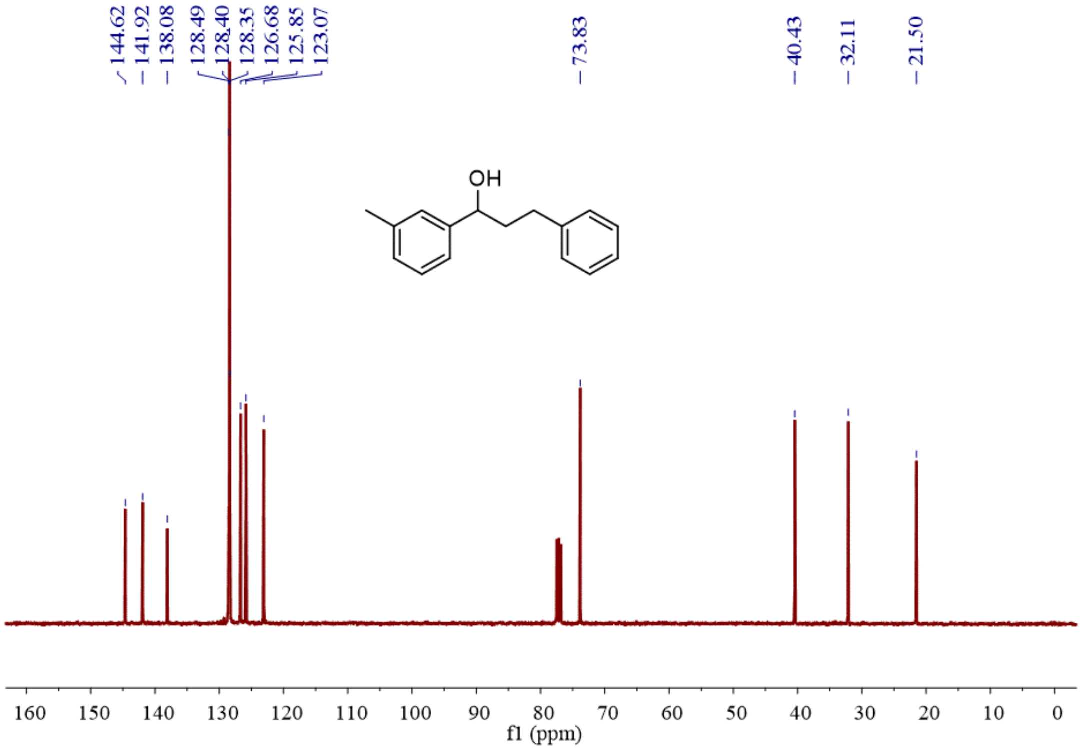

Figure S38. ^{1}H and ^{13}C NMR spectra of **3ka**

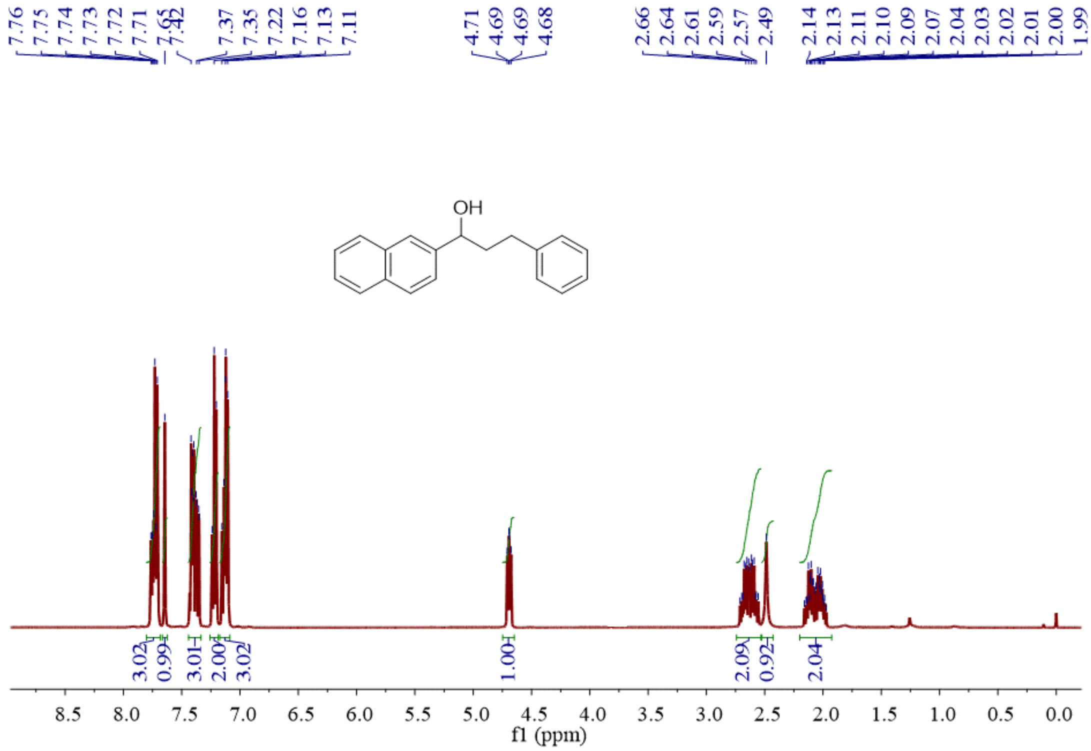

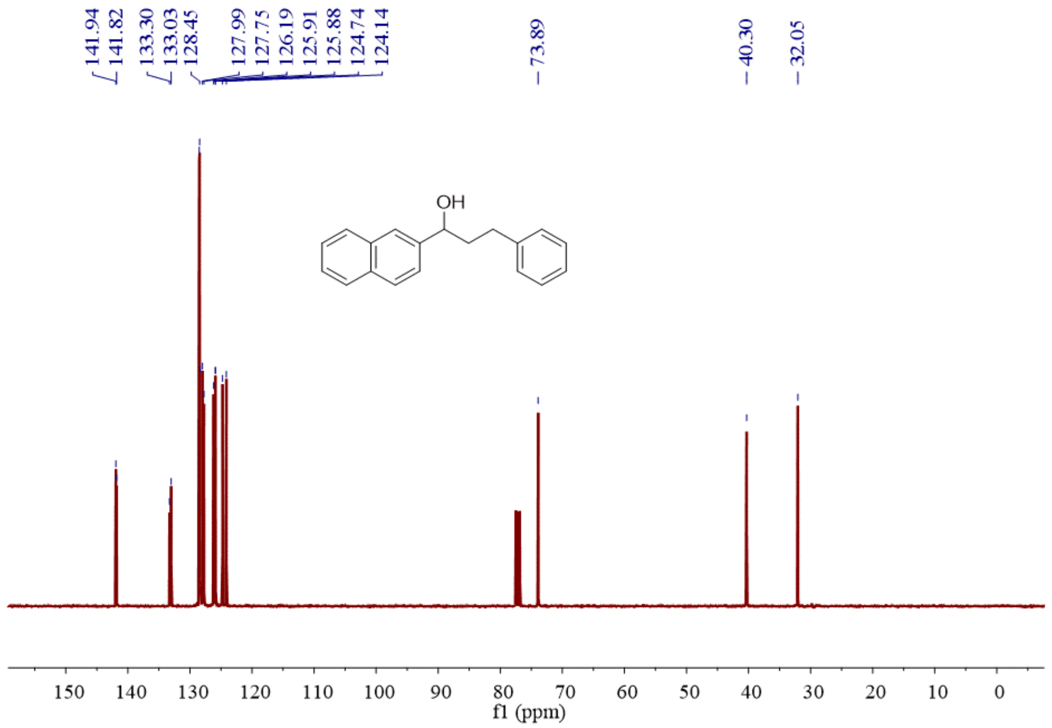

Supplement: Supplementary file 1 [file polymers-14-00231-s001.zip › polymers-1535629-supplementary.pdf]
